# Genomic approaches to identify and investigate genes associated with atrial fibrillation and heart failure susceptibility

**DOI:** 10.1186/s40246-023-00498-0

**Published:** 2023-06-03

**Authors:** Kush Ketan Patel, Cynthia Venkatesan, Habiba Abdelhalim, Saman Zeeshan, Yuichiro Arima, Suvi Linna-Kuosmanen, Zeeshan Ahmed

**Affiliations:** 1 Rutgers Institute for Health, Health Care Policy and Aging Research, Rutgers University, 112 Paterson St, New Brunswick, NJ USA; 2 Rutgers Cancer Institute of New Jersey, Rutgers University, 195 Little Albany St, New Brunswick, NJ USA; 3 Developmental Cardiology Laboratory, International Research Center for Medical Sciences, Kumamoto University, 2-2-1 Honjo, Kumamoto City, Kumamoto Japan; 4 A. I. Virtanen Institute for Molecular Sciences, University of Eastern Finland, 70211 Kuopio, Finland; 5 Computer Science and Artificial Intelligence Laboratory, Massachusetts Institute of Technology, Cambridge, MA 02139 USA; 6 Broad Institute of MIT and Harvard, Cambridge, MA 02142 USA; 7 Department of Genetics and Genome Sciences, UConn Health, 400 Farmington Ave, Farmington, CT USA; 8 Department of Medicine, Robert Wood Johnson Medical School, Rutgers Biomedical and Health Sciences, 125 Paterson St, New Brunswick, NJ USA

**Keywords:** Genes, Genetic loci, Heart failure, Atrial fibrillation, Cardiovascular diseases, Genomics, Multi-OMICS

## Abstract

Atrial fibrillation (AF) and heart failure (HF) contribute to about 45% of all cardiovascular disease (CVD) deaths in the USA and around the globe. Due to the complex nature, progression, inherent genetic makeup, and heterogeneity of CVDs, personalized treatments are believed to be critical. To improve the deciphering of CVD mechanisms, we need to deeply investigate well-known and identify novel genes that are responsible for CVD development. With the advancements in sequencing technologies, genomic data have been generated at an unprecedented pace to foster translational research. Correct application of bioinformatics using genomic data holds the potential to reveal the genetic underpinnings of various health conditions. It can help in the identification of causal variants for AF, HF, and other CVDs by moving beyond the one-gene one-disease model through the integration of common and rare variant association, the expressed genome, and characterization of comorbidities and phenotypic traits derived from the clinical information. In this study, we examined and discussed variable genomic approaches investigating genes associated with AF, HF, and other CVDs. We collected, reviewed, and compared high-quality scientific literature published between 2009 and 2022 and accessible through PubMed/NCBI. While selecting relevant literature, we mainly focused on identifying genomic approaches involving the integration of genomic data; analysis of common and rare genetic variants; metadata and phenotypic details; and multi-ethnic studies including individuals from ethnic minorities, and European, Asian, and American ancestries. We found 190 genes associated with AF and 26 genes linked to HF. Seven genes had implications in both AF and HF, which are *SYNPO2L, TTN, MTSS1, SCN5A, PITX2, KLHL3*, and *AGAP5*. We listed our conclusion, which include detailed information about genes and SNPs associated with AF and HF.

**Supplementary Information:**

The online version contains supplementary material available at 10.1186/s40246-023-00498-0.

## Introduction

Cardiovascular disease (CVD) is one of the major contributors to morbidity and mortality in the USA and even around the globe [1]. In recently reported studies, the overall lifetime risk associated with CVD does not differ significantly between males and females as variable cardiovascular risk factors (e.g., systolic blood pressure, high-density lipoprotein cholesterol, hemoglobin A1c, and smoking behavior) were found to be similar [2]. Additionally, estrogens and androgens produced by gonadal hormones have been proven to play an important role for sex differences in the occurrence and progression of CVDs. Even though the lifetime risk is similar between the sexes, men tend to develop CVDs before women [2]. This difference in the manifestation of CVD between the sexes is accounted for by the roles that sex chromosomal mechanisms have in the control of diseases. Atrial fibrillation (AF) and heart failure (HF) are among the most common manifestations of CVDs [3]. AF is an arrhythmic disorder in the atrium of the heart, which can cause irregular heart rhythms [4], whereas HF is a chronic disorder, which weakens heart muscles and affects the regular function of the heart to pump enough oxygen-rich blood [5]. Hypertension, diabetes, obesity, and valvular heart disease are among the known risk factors for AF and HF [6].

We are only in the developing stages of identifying causes of end-stage CVDs and providing personalized treatments with predictive analysis and deep phenotyping. Despite significant advancements in CVD diagnostics, prevention, and treatment, approximately half of the affected CVD patients reportedly die within five years of receiving a diagnosis [7, 8]. CVDs have a complex multifactorial etiology involving both genetic and environmental agents [9]. Evidence from the Framingham Heart Study suggests that there is a genetic component related to CVDs including AF and HF [10–12]. Due to the advancements in sequencing technologies, genomic data are developing at an unmatched pace and at levels to foster translational research. Different types of technologies are available today for sequencing the genome and transcriptome of patients with common, complex, and rare disorders including CVDs. Among the most popular and widely used are Illumina sequencing for single and pair end short reads, and Oxford Nanopore and PacBio for long-read sequencing [13]. Using these technologies, heterogeneous sequence data can be produced, which includes but are not limited to whole-genome sequencing (WGS), whole-exome sequencing (WES), RNA sequencing (RNA-seq), single-cell RNA sequencing (scRNA-seq), chromatin immunoprecipitation followed by sequencing (ChIP–seq), assay for transposase-accessible chromatin with sequencing (ATAC-seq), etc. The effective downstream analysis of these data types has demonstrated a strong biological correlation in multiple CVD studies [14]. Genomic information, including high-quality sequenced DNA and RNA-seq of transcribed genes, informs us of a CVD patient’s inherent genetic makeup with the most comprehensive view of the genome [15, 16]. DNA-based gene variant detection when combined with RNA-seq-driven gene expression analysis has the potential to reveal novel and sensitive biomarkers and stratify CVD patient populations based on their disease risk [17]. It can also assist in advancing our understanding of CVD biology as a variety of biological processes, including gene regulatory mechanisms, are influenced by a complex interplay of environmental and genetic factors that shape the onset and clinical course of AF, HF, and other CVDs [18, 19].

Over ten million genomic datasets have been produced and publicly shared in the year 2022. Public data repositories and forums include but are not limited to the NCBI Sequence Read Archive (SRA) [20]; the Encyclopedia of DNA Elements (ENCODE) [21]; the Genotype-Tissue expression (GTEx) project [22]; UK BioBank [23]; the Functional Annotation of Animal Genomes project (FAANG) [24]; and Earth BioGenomes Project (EBP) [25]. The GTEx project has one of the most comprehensive gene expression datasets presently available. This repository was created to identify genetic variations linked with alterations in gene expression [26]. GTEx also encompasses pathology and histology information along with additional attributes such as ethnicity, age, and gender. ENCODE has detected and annotated a substantial number of functional elements in the human genome [26]. Diverse and high-volume genomic and phenotypic data available through these platforms have the potential to broaden the scope of biological discoveries and insights by extracting, analyzing, and interpreting the hidden information [27]. The incorporation of diverse data frameworks could also be immensely effective in comprehending the mechanisms involved in the manifestation of diseases. Correct application of the standard genomics approaches holds the potential to decipher the genetic underpinnings for a variety of health conditions. Based on the variable sequence data types, customized downstream analyses can be performed utilizing variable bioinformatics tools and technologies [14]. Most common examples include but are not limited to gene expression, enrichment, pathway, functional/non mutation, and different kinds of statistical analyses [28]. In this study, we are focused on examining the state-of-the-art genomic approaches to investigate genes associated with CVDs, with special emphasis on AF (Table 1) and HF (Table 3) as well as the common genes for both AF and HF (Table 5).

**Table 1:** Genes associated with Atrial Fibrillation (AF)

#	Gene(s)	SNP rsID	Chromosome	Gene location	PMID
1	*UBE4B*	rs187585530	1	Missense	29892015
2	*CASZ1*	rs880315; rs284277	1	Intron	29892015; 30061737
3	*HSPG2*	rs7529220	1	Regulatory region	30061737
4	*CELA3B*	rs7529220	1	Regulatory region	30061737
5	*SCMH1*	rs2885697	1	Intronic	30061737
6	*AGBL4*	rs11590635	1	Intronic	30061737
7	*C1orf185*	rs146518726	1	Intron	29892015; 30061737
8	*KCND3*	rs12044963; rs1545300	1p13.2	Intron	29892015/ 28416822; 30061737
9	*CASQ2*	rs4484922; rs4073778	1	Intron	29892015; 30061737
10	*GJA5*	rs79187193	1	Upstream gene	29892015; 30061737
11	*KCNN3*	rs11264280	1	Intergenic	29892015; 30061737
12	*PMVK*	rs11264280	1	Intergenic	29892015; 30061737
13	*METTL11B*	rs72700114; rs72700118	1; 1q24	Intergenic	29892015/ 30061737; 28416818
14	*KIFAP3*	rs72700118	1q24	Intergenic	28416818
15	*LINC01142*	rs72700114	1	Intergenic	29892015; 30061737
16	*GORAB*	rs608930	1	Intergenic	29892015
17	*PRRX1*	rs608930; rs3903239	1; 1q24	Intergenic; 46 kb upstream	29892015; 22544366
18	*PPFIA4*	rs10753933; rs17461925	1; 1q32.1	Intron	29892015/ 30061737; 28416822
19	*NUCKS1*	rs4951261; rs4951258	1	Intron	29892015; 30061737
20	*NPPA*	N/A	1p36.22	N/A	33950154
21	*KIF3C*	rs6546620; rs7578393	2	Intron	29892015; 30061737
22	*XPO1*	rs6742276; rs11125871	2	Upstream gene	29892015; 30061737
23	*CEP68*	rs2540949; rs2540953	2p14; 2	Intronic	28416818/ 29892015/ 30061737; 28416822
24	*SLC1A4*	rs2540953	2	N/A	28416822
25	*SNRNP27*	rs10165883; rs6747542	2	Upstream gene	29892015; 30061737
26	*REEP1*	rs72926475	2	Intergenic	29892015; 30061737
27	*KDM3A*	rs72926475	2	Intergenic	29892015; 30061737
28	*GYPC*	rs28387148	2	Intronic	30061737
29	*TEX41*	rs67969609	2	Intronic	30061737
30	*MBD5*	rs12992412	2	Intron	29892015
31	*WIPF1*	rs56181519	2	Intergenic	29892015; 30061737
32	*CHRNA1*	rs56181519	2	Intergenic	29892015; 30061737
33	*TTN*	rs35504893; rs2288327	2; 2q31	Intronic	29892015; 28416818/ 30061737/ 33950154
34	*TTN-AS1*	rs2288327	2q31	Intronic	28416818
35	*SPATS2L*	rs295114; rs3820888	2	Intron	29892015; 30061737
36	*ERBB4*	rs35544454	2	Intronic	30061737
37	*ANXA4*	rs3771537	2p13	Intronic	28416818
38	*GMCL1*	rs3771537	2p13	Intronic	28416818
39	*CAND2*	rs6810325; rs4642101; rs7650482	3; 3p25	Intronic	29892015; 25124494; 30061737
40	*THRB*	rs73032363; rs73041705	3	Intron	29892015; 30061737
41	*SCN10A*	rs6790396; rs6800541	3; 3p22	Intronic	29892015/ 30061737; 28416818; 33950154
42	*SCN5A*	N/A	3p22.2	N/A	33950154
43	*LRIG1*	rs2306272; rs34080181	3	Missense	29892015; 30061737
44	*FRMD4B*	rs17005647	3	Intronic	30061737
45	*EPHA3*	rs7632427; rs6771054	3	Downstream gene	29892015; 30061737
46	*PHLDB2*	rs17490701; rs10804493	3	Intron	29892015; 30061737
47	*PPP2R3A*	rs1278493	3	Intronic	30061737
48	*GNB4*	rs4855075; rs7612445	3	Upstream gene	29892015; 30061737
49	*XXYLT1*	rs60902112	3	Intronic	30061737
50	*PAK2*	rs9872035	3	Intron	29892015
51	*WDR1*	rs3822259	4	Upstream gene	29892015
52	*PRDM8*	rs1458038	4	Intergenic	30061737
53	*FGF5*	rs1458038	4	Intergenic	30061737
54	*SLC9B1*	rs3960788; rs10006327	4	Intron	29892015; 30061737
55	*PITX2*	rs2129977; rs67249485; rs17042171	4	Intergenic	29892015; 30061737; 19597492
56	*C4orf32*	rs2129977; rs67249485	4	Intergenic	29892015; 30061737
57	*CAMK2D*	rs55754224; rs6829664	4	Intron	29892015; 30061737
58	*ARHGAP10*	rs10213171	4	Intron	29892015; 30061737
59	*HAND2*	rs10520260; rs7698692; rs12648245	4; 4q34.1	Downstream gene	29892015; 28416822; 30061737
60	*LOC102467213*	rs6596717	5	Intergenic	30061737
61	*EFNA5*	rs6596717	5	Intergenic	30061737
62	*KCNN2*	rs716845; rs337711; rs337705	5; 5q22	Intronic	29892015; 28416818; 30061737
63	*FBN2*	rs2012809	5	Intergenic	30061737
64	*SLC27A6*	rs2012809	5	Intergenic	30061737
65	*WNT8A*	rs34750263; rs2967791; rs2040862	5; 5q31	Intergenic; intronic	29892015; 28416818; 30061737
66	*NME5*	rs34750263; rs2040862	5	Intergenic	29892015; 30061737
67	*ARHGAP26*	rs174048; rs6580277	5	Intergenic	29892015; 30061737
68	*NR3C1*	rs174048; rs6580277	5	Intergenic	29892015; 30061737
69	*SLIT3*	rs12188351	5	Intronic	30061737
70	*NKX2-5*	rs6882776; rs6891790	5	Upstream gene	29892015; 30061737
71	*KLHL3*	rs2967791	5q31	Intronic	28416818
72	*FAM13B*	rs2967791	5q31	Intronic	28416818
73	*ATXN1*	rs73366713	6	Intron	29892015; 30061737
74	*KDM1B*	rs34969716	6	Intron	29892015; 30061737
75	*C6orf1*	rs1307274	6	Regulatory region	29892015
76	*NUDT3*	rs1307274	6	Regulatory region	29892015
77	*CDKN1A*	rs3176326	6	Intron	29892015; 30061737
78	*CGA*	rs6907805; rs2031522	6	Intergenic	29892015; 30061737
79	*ZNF292*	rs6907805; rs2031522	6	Intergenic	29892015; 30061737
80	*GOPC*	rs210632	6	Downstream gene	29892015
81	*SLC35F1*	rs17079881; rs4946333; rs89107; rs3951016	6; 6q22; 6q22	Intronic	29892015;28416818; 28416818; 30061737
82	*PLN*	rs4946333; rs89107	6q22	Intronic	28416818; 28416818
83	*GJA1*	rs13191450; rs13216675; rs13195459	6; 6q22	Intergenic	29892015; 25124494; 30061737
84	*HSF2*	rs13191450; rs13195459	6	Intergenic	29892015; 30061737
85	*LINC00326*	rs12208899	6	Intergenic	29892015
86	*EYA4*	rs12208899	6	Intergenic	29892015
87	*UST*	rs117984853	6	Downstream gene	29892015; 30061737
88	*SUN1*	rs11768850	7	Intron	29892015
89	*DGKB*	rs55734480	7	Intron	29892015; 30061737
90	*CREB5*	rs6462078; rs6462079	7	Intron	29892015; 30061737
91	*GTF2I*	rs74910854; rs35005436	7	Intron	29892015; 30061737
92	*CDK6*	rs11773884; rs56201652	7	Intron	29892015; 30061737
93	*COG5*	rs62483627	7	Intron	29892015
94	*CAV1*	rs11773845; rs3807989	7; 7q31	Intronic	29892015/ 30061737; 22544366
95	*OPN1SW*	rs55985730	7	Upstream gene	29892015; 30061737
96	*KCNH2*	rs7789146	7	Intron	29892015; 30061737
97	*LINC00208*	rs35620480	8	Regulatory region	30061737
98	*GATA4*	rs35620480	8	Regulatory region	30061737
99	*ASAH1*	rs7508	8; 8p22	3' utr	29892015; 28416818; 30061737
100	*PCM1*	rs7508	8p22	3′ utr	28416818
101	*XPO7*	rs7846485; rs7834729	8	Intron	29892015; 30061737
102	*FBXO32*	rs62521286	8	Intron	29892015; 30061737
103	*MTSS1*	rs35006907	8	Regulatory region	29892015
104	*LINC00964*	rs35006907	8	Regulatory region	29892015
105	*MIR30B*	rs7460121	8	Downstream gene	29892015
106	*PTK2*	rs6993266; rs6994744	8	Intron	29892015; 30061737
107	*SLC24A2*	rs4977397	9	Intergenic	29892015
108	*MLLT3*	rs4977397	9	Intergenic	29892015
109	*C9orf3*	rs4385527; rs10821415	9	Intron	29892015; 30061737
110	*C9orf3 (FBP1)*	rs10821415	9q22	Intronic	22544366
111	*C9orf3 (FBP2)*	rs10821415	9q22	Intronic	22544366
112	*ZNF462*	rs4743034	9	Intron	29892015
113	*PSMB7*	rs10760361	9	Upstream gene	29892015
114	*LHX3*	rs2274115	9	Intronic	30061737
115	*REEP3*	rs7919685; rs12245149	10	Intron	29892015; 30061737
116	*SIRT1*	rs7096385	10	Intronic	30061737
117	*SYNPO2L*	rs60212594; rs10824026	10; 10q22	Intron; 5 kb upstream	29892015/ 30061737; 22544366
118	*MYOZ1*	rs10824026	10q22	20 kb upstream	22544366
119	*C10orf11*	rs11001667; rs10458660	10	Intron	29892015; 30061737
120	*C10orf76*	rs1044258	10	3' utr	29892015
121	*NEURL*	rs11598047; rs12415501; rs6584555	10; 10q24; 10q24	Intronic	29892015/ 30061737; 25124494; 25124494
122	*RBM20*	rs10749053	10	Intronic	30061737
123	*SH3PXD2A*	rs35176054, rs202011870; rs2047036	10q24; 10q24; 10q24.33	Intronic	28416818; 28416818; 28416822
124	*NEBL*	rs2296610	10p12	Missense variant	28416822
125	*NAV2*	rs1822273; rs10741807	11	Intron	29892015; 30061737
126	*SORL1*	rs949078; rs4935786	11	Intergenic	29892015; 30061737
127	*MIR100HG*	rs949078; rs4935786	11	Intergenic	29892015; 30061737
128	*KCNJ5*	rs76097649; rs75190942	11; 11q24	Upstream gene; intronic	29892015/ 30061737; 28416818
129	*KCNQ1*	N/A	11p15.5-p15.4	N/A	33950154
130	*KCNJ11*	N/A	11p15.5	N/A	33950154
131	*LINC00477*	rs10842383; rs4963776	12	Intergenic	29892015; 30061737
132	*BCAT1*	rs10842383; rs4963776	12	Intergenic	29892015; 30061737
133	*SSPN*	rs113819537; rs17380837	12	Upstream gene	29892015; 30061737
134	*PKP2*	rs12809354	12	Intron	29892015; 30061737
135	*NACA*	rs7978685; rs2860482	12	Downstream gene	29892015; 30061737
136	*BEST3*	rs35349325; rs71454237	12	Upstream gene	29892015; 30061737
137	*KRR1*	rs11180703; rs12426679	12	Intergenic	29892015; 30061737
138	*PHLDA1*	rs11180703; rs12426679	12	Intergenic	29892015; 30061737
139	*TBX5*	rs883079; rs10507248	12; 12q24	3' utr; intronic	29892015/ 30061737; 25124494
140	*CUX2*	rs6490029	12q24	Intronic	25124494
141	*TBX5-AS1*	rs12810346	12	Intergenic	29892015
142	*TBX3*	rs12810346	12	Intergenic	29892015
143	*HIP1R*	rs10773657	12	Intronic	30061737
144	*DNAH10*	rs12298484	12	Intron	29892015
145	*FBRSL1*	rs6560886	12	Intronic	30061737
146	*SOX5*	rs11047543	12p12	Intergenic	28416818
147	*CACNA1C*	N/A	12p13.33	N/A	33950154
148	*LINC00540*	rs9580438; rs9506925	13	Intergenic	29892015; 30061737
149	*BASP1P1*	rs9580438; rs9506925	13	Intergenic	29892015; 30061737
150	*CUL4A*	rs35569628	13	Intronic	30061737
151	*MYH7*	rs28631169; rs422068	14	Intron	29892015; 30061737
152	*AKAP6*	rs2145587; rs11156751	14	Intron	29892015; 30061737
153	*SNX6*	rs73241997	14	Intergenic	29892015; 30061737
154	*CFL2*	rs73241997	14	Intergenic	29892015; 30061737
155	*SYNE2*	rs2738413; rs1152591	14; 14q23	Intronic	29892015/ 30061737; 22544366
156	*DPF3*	rs74884082	14	Intronic	30061737
157	*LRRC74*	rs10873299; rs10873298	14	Intergenic	29892015; 30061737
158	*IRF2BPL*	rs10873299; rs10873298	14	Intergenic	29892015; 30061737
159	*MYH6*	N/A	14q11.2	N/A	33950154
160	*GCOM1*	rs147301839	15	Missense	30061737
161	*USP3*	rs62011291; rs7170477	15	Intron	29892015; 30061737
162	*TLE3*	rs12591736	15	Intergenic	29892015
163	*UACA*	rs12591736	15	Intergenic	29892015
164	*HCN4*	rs74022964; rs7164883	15; 15q24	Intergenic; intronic	29892015/ 30061737; 22544366
165	*REC114*	rs74022964	15	Intergenic	29892015; 30061737
166	*LINC00927*	rs12908004	15	Intron	29892015; 30061737
167	*ARNT2*	rs12908004	15	Intron	29892015; 30061737
168	*IGF1R*	rs12908437; rs4965430	15	Intron	29892015; 30061737
169	*RPS2*	rs2286466; rs140185678	16	Synonymous	29892015; 30061737
170	*ZFHX3*	rs2359171; rs2106261	16; 16q22	Intronic	29892015/ 30061737; 19597492
171	*YWHAE*	rs7225165	17	Intergenic	30061737
172	*CRK*	rs7225165	17	Intergenic	30061737
173	*POLR2A*	rs8073937; rs9899183	17	Intergenic	29892015; 30061737
174	*TNFSF12*	rs8073937; rs9899183	17	Intergenic	29892015; 30061737
175	*MYOCD*	rs72811294	17	Intron	29892015; 30061737
176	*ZPBP2*	rs11658278	17	Intronic	30061737
177	*MAPT*	rs242557	17	Intron	29892015
178	*GOSR2*	rs76774446; rs1563304	17	Downstream gene	29892015; 30061737
179	*KCNJ2*	rs7219869	17	Intergenic	29892015
180	*CASC17*	rs7219869	17	Intergenic	29892015
181	*CYTH1*	rs12604076	17	Intronic	30061737
182	*SMAD7*	rs9953366	18	Intron	29892015; 30061737
183	*MEX3C*	rs8088085	18	Intronic	30061737
184	*CASC20*	rs2145274	20	Regulatory region	29892015
185	*BMP2*	rs2145274	20	Regulatory region	29892015
186	*C20orf166*	rs7269123	20	Intron	29892015
187	*LOC100506385*	rs2834618	21	Intron	29892015; 30061737
188	*TUBA8*	rs465276; rs464901	22	Intron	29892015; 30061737
189	*MYO18B*	rs133902	22	Splice region	30061737
190	*KCNE5*	N/A	Xq23	N/A	33950154

This table includes information about genes, SNP, chromosome, location, and article reported

Understanding the genetic basis of complex CVDs can improve genetic risk scoring which can now outperform traditional risk factors in risk prediction [28, 29]. Along with genetic factors, familial diabetes, high blood pressure, or high cholesterol can also have genetic predisposition to elevated risk of CVD [30]. Estimating heritability is a measure to compute the genetic variant influence and familial aggregation in the CVD population [31, 32]. CVD is a highly heritable disease with genetic traits that may have monogenic or polygenic backgrounds. Approximately 40% of the CVD risk lies in hereditary factors [33]. Of these factors, monogenetic conditions can lead to severe premature CVD and early death if unrecognized and untreated [34]. In past years, attempts have been made to uncover the connections between non-coding active elements of the genome and enhancers and their significance on disease pathology [26]. Now, further advances in next-generation sequencing have triggered increased attention on interpreting variants in genes associated with heritable CVDs. Genome-wide associations study (GWAS) has led to the detection of some of the most powerful genetic determinants of CVD, mostly single nucleotide polymorphisms of unambiguous and high importance that modify cardiomyopathy. Although GWAS provides vital information, SNP array-based GWAS lacks the understanding of ultra-rare variants associated with diseases making it difficult to predict the heritability of complex traits, which are a mix of common variants, rare and ultra-rare variants. Therefore, GWAS offers little or negligible molecular evidence of gene causality [26]. However, implementing GWAS with a large WGS reference panel is an alternative solution to obtaining information with a bit more precision and eliminating many limitations observed in the usage of SNP arrays solely. In this study, we have reviewed and compared the related high-quality scientific literature published between 2009 and 2022 that is accessible through PubMed. While selecting relevant literature, we were mainly focused on identifying possible recent genomic approaches involving but not limited to the integration of genomic data; analyses of common and rare genetic variants; meta- and phenotypic analysis; and multi-ethnic studies including individuals from ethnic minorities, European, Asian, and American ancestries. Additional information regarding individual articles’ sample population size, cohort age, sex, ethnic group, clinical characteristics, methodological approaches, P-values, and type of study is reported for AF (Table 2) and HF (Table 4).

**Table 2:** Article details about genes associated with Atrial Fibrillation (AF)

PMID	Genes#	Sample Size	Ethnicity	Traits	P-values	NGS Type	Analysis
28416818	12	GWAS: AF = 17,931 Referents = 115,142 ExWAS, RVAS: AF = 22,346 Referents = 132,086	Multi-ethnic: European, Asian, African-American, Brazilian, Hispanic	hypertension, myocardial infarctions, diabetes mellitus, heart failure	P < 5E − 8 (GWAS); P < 1.04E − 6 (ExWAS); P = 3.3E − 11 (RVAS)	WGS, WES	SNPtest, ProABE, and PLINK, seqMeta package of R statistical power
25,124494	5	GWAS: European: AF = 6,691 Referents = 17,142 Japanese: AF = 8,373 Referents = 20,540	European, Japanese	diabetes, myocardial infarctions, congestive heart failure	P < 5E5	N/A	N/A
33950154	9	Male = 132 Female = 95	African, Hispanic	hypertension, type II diabetes, congestive heart failure, coronary artery disease, valve heart disease, left ventricular ejection fraction, vascular disease	P ≤ 5.0E-2	Illumina NextSeq500, Sanger sequencing	N/A
19597492	1	GWAS: European: AF = 896 Referents = 15,768 European-American: AF = 2,517 Referents = 21,337	European, European-American	prevalent AF after CKD diagnosis, incident AF after CKD diagnosis	P = 2.3E − 7	N/A	ProbABEL, R ProbABEL, PLINK, R R, version 2.7 PLINK, R Mach2QTL GenABEL + PLINK
22544366	6	GWAS: AF = 6,707 Controls = 52,426	European, Japanese	hypertension, diabetes, heart failure, myocardial infarction	P < 5E − 8	N/A	ProbABEL, R, PLINK, R packages kinship, GEE, COXPH, Mach2QTL GenABEL + PLINK, GRIMP, QUICKTEST v0.94, PLATO, R, version 2.10
28416822	6	GWAS: AF = 8,180 Controls = 28,612	Japanese	diabetes, myocardial infarction, heart failure	P < 5.0E − 8	N/A	N/A
29892015	97 (67 novel)	GWAS: AF = 65,446	Multi-ethnic: European, Japanese, African-American, Brazilian, Hispanic	hypertension, diabetes mellitus, myocardial infarction, heart failure	P < 1E − 8	RNA-seq	ProbABEL, SNPTEST, FAST, mach2dat, R, EPACTS, Hail, and PLINK
30061737	111 (80 novel)	GWAS: AF = 60,620 Controls = 970,216	Multi-ethnic	N/A	P < 5E − 8	N/A	N/A

This table includes information about article reported, total number of genes, sample size, ethnicity, clinical traits, P-values, next-generation sequencing (NGS) type, and analysis performed using different tools

## Multi-omics approaches investigating genes associated with atrial fibrillation (AF)

**Fig. 1:**
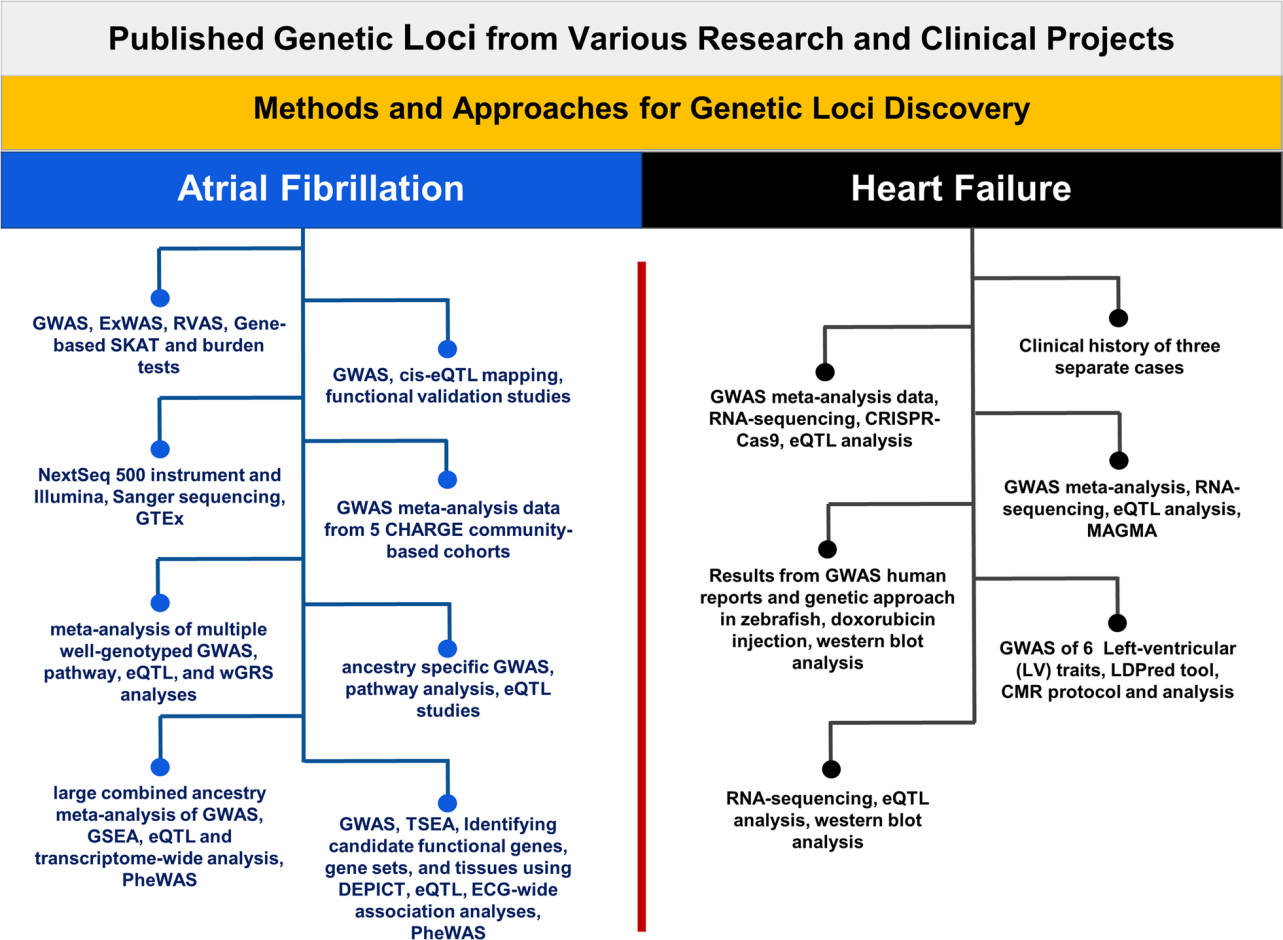
Genomics approaches for genetic loci discovery

In this section, a review of literature outlines a detailed genetic understanding and etiology of AF and genetic loci discovery (Fig. 1).

### Large-scale analyses of common and rare variants identify new loci associated with AF [35]

Prior to the study by Christophersen et al*.*, 14 genetic loci in relation to AF existed [36–40]. Christophersen et al*.* performed GWAS, exome-wide association studies (ExWAS), and rare variant association studies (RVAS) to determine 12 novel AF loci and further develop the underlying genetic basis of AF [35]. This multi-ethnic meta-analysis of rare and common variants involved genes in cardiac, electrical, and structural remodeling. The genetic variants for AF were determined by investigating a large population of individuals from the AF Genetics (AFGen) Consortium. All meta-analyses combined involved 22,346 individuals with AF as well as 132,086 controls who did not present AF at time of sample collection. The GWAS study included 17,931 individuals with AF and 115,142 controls. Together, the ExWAS and RVAS studies had 22,346 individuals with AF and 132,086 controls. The participants’ ages ranged from 18 to 100, while the mean ages for most of the cohorts were from 50 tp 77. Coming from a variety of ancestral groups, the participants were of European, Asian, African-American, Hispanic, and Brazilian descent, with a large part of the cases being European. Some also presented with pre-existing conditions that included hypertension, myocardial infarction, diabetes mellitus, and heart failure.

For the GWAS meta-analyses, genotyping was performed on every study, which was then added to the 1000 Genomes Project Phase One reference panel. The AF cases, both prevalent and incidental, underwent two analyses. First, at blood draw, those who presented with AF were deemed prevalent cases, and those without AF were considered referent cases. This was done using logistic regression analysis. Second, for incidental cases, Cox proportional-hazard models were used to analyze the time of AF diagnosis. The inverse-variance-weighted fixed-effects model with Meta-Analysis Helper (METAL) was used to analyze the GWAS results. All ExWAS and RVAS studies had undergone exome variant genotyping. This was done in conjunction with local association analyses which were performed with a logistic model. This model puts together all the cases including prevalent, incident, and controls. SKAT and burden tests were performed on the RVAS data of rare variants from the exome chip array. The software GCTA was implemented for the approximate joint and conditional association analysis. Expression quantitative trait loci analyses (eQTLs) were performed using samples of human left atrial tissue, and the eQTL examination was conducted with the aid of the GTEx database. To support the findings of the discovery study, the results were validated using the BioBank Japan Study and the UK BioBank Study. To correlate causal genes to the genetic loci from the discovery GWAS, the DEPICT analysis was utilized. Data analysis was subjected to Qiagen’s Ingenuity Pathway Analysis, after which the location of the genetic variants was examined.

Ten novel genetic loci were identified to be linked to AF through the GWAS. The genes and single nucleotide polymorphisms (SNPs) included: *METTL11B/KIFAP3* with SNP rs72700118 on chromosome 1q24, *ANXA4/GMCL1* with SNP rs3771537 on chromosome 2p13, *CEP68* with SNP rs2540949 on chromosome 2p14, *TTN/TTN/AS1* with SNP rs2288327 on chromosome 2q31, *KCNN2* with SNP rs33771 on chromosome 5q22, *KLHL3/WNT8A/FAM13B* with SNP rs2967791 on chromosome 5q31, *SLC35F1/PLN* with SNP rs4946333 on chromosome 6q22, *ASAH1/PCM1* with SNP rs7508 on chromosome 8p22, *SH3PXD2A* with SNP rs35176054 on chromosome 10q24, and *KCNJ5* with SNP rs75190942 on chromosome 11q24. Two new genetic loci were also discovered through the ExWAS study that included genes *SCN10A* with SNP rs6800541 on chromosome 3p22 and *SOX5* with SNP rs11047543 on chromosome 12p12. Another variant, SNP rs89107, was found on gene *SLC35F1/PLN*, a genetic locus also discovered in the GWAS study. Various electrocardiographic traits are linked to these three variants from the ExWAS studies. In the RVAS study, the gene *SH3PXD2A,* previously mentioned in the GWAS studies*,* was found to be associated with AF by a rare coding variant, rs202011870. The role of this gene is to encode a tyrosine kinase substrate, *TKS5,* which has cardiac expression in the atria and ventricles. In relation to cancer, this substrate plays a role in determining how invasive the cancer cells may be [41], and in relation to Alzheimer’s disease, alongside the gene *ADAM12*, it moderates the neurotoxicity of β-amyloid [42]. Even though the role of *SH3PXD2A* is established in these other two major diseases, its role in AF is still unclear.

This association between SNP rs202011870 and gene *SH3PXD2A* is unique to those of Asian ancestry; however, there was no genome-wide significance for variants that may be linked to African-American or European individuals in the RVAS analyses. These genes, *METTL11B/KIFAP3, ANXA4/GMCL, CEP68, TTN/TTN/AS1, KCNN2, KLHL3/WNT8A/FAM13B, SLC35F1/PLN, ASAH1/PCM1, SH3PXD2A, KCNJ5, SCN10A,* and *SOX5*, related to the twelve new genetic loci identified in this study generally encode for transcription factors, ion channels, and sarcomeric proteins. Ion channels, specifically sodium and potassium channels, are encoded by five of these genes. As a result, two of these genes, *KCNN2* and *KCNJ5,* have developed an association with the action potential of the atria [43]. *KCNN2* encodes the channel SK2, a calcium-dependent potassium channel [43]. This creates a heteromeric channel complex with the SK3 protein produced by a gene reported to be associated with AF in a previous GWAS study, the *KCNN3* gene [38, 40]. The *KCNJ5* gene helps form the IKACh channel complex by encoding Kir3.4, a potassium channel [43]. This produces heteromers with Kir3.1. The IKACh channel complex has been previously noted to be a target for AF therapies [44]. Numerous AF loci were found to be in relation to genes that encode for transcription factors that include *PITX2, ZFHX3, PRRX1, SOX5*, and *TBX5*. Of the genetic loci (novel and known) found to be associated with AF in this study, seven were related to several AF phenotypes that include electrocardiographic traits, stroke, and left ventricle internal diastolic diameter. More specifically, the genes *PITX2/C4orf32* and *ZFHX3* possessed a relation to stroke, while the genes *KLHL3/WNT8A, HCN4/ C15orf60,* and *SLC35F1/PLN* were found to be phenotypically associated with heart rate. The *SLC35F1/PLN* genes demonstrated association with QRS, QT, and echo left ventricle internal diastolic diameter phenotypes. The *CAV1* and *TBX5* genes were found related to the PR-I, QRS, and QT phenotypes and *CAV1* also exhibited a relation to PR-S.

Although the study accumulated many strengths, the authors mentioned some limitations. AF is a qualitative trait that can make measuring genetic variation difficult. One reason for this was that each age group had inconsistency in the AF prevalence. According to Christophersen et al*.,* all the genetic loci discovered in this study only portray a small amount of the genetic variance associated with AF.

### Integrating genetic, transcriptional, and functional analyses to identify novel genes for AF [36]

This study performed by Sinner et al*.* discovered a total of five novel genetic loci for AF [36]. Using genotyping, eQTL studies, and functional validation, four novel loci in Europeans were identified and two novel loci in Japanese individuals, one overlapping between both ethnic groups. The cohorts included both prevalent and incidental AF cases. The discovery genome-wide association study included 6707 European cases with 52,426 European controls and 843 Japanese cases with 3350 Japanese controls. The subjects were over 30 years of age, and some presented with other underlying conditions which included diabetes, myocardial infarction, and congestive heart failure. To perform replication studies of the results curated from the initial GWAS, 6691 European AF cases and 17,144 controls were accumulated through nine studies from various cohorts that had previous GWAS data or genotyping performed. Of the AF cases, two-thirds were males, and the mean age was 64.2 ± 8.3 years, while the mean age of the controls was 66.1 ± 7.9 years with approximately half of them males. For the Japanese GWAS, two replication studies were performed that involved 1618 Japanese AF cases and 17,190 control cases as an initial dataset, and a secondary dataset of 5912 Japanese AF cases. AFGen consortium genome-wide associations datasets were analyzed to identify genetic variants for AF. The selection process included determining those SNPs that had associations to AF (using a meta-analysis value of P < 5 × 10–5), analyzing the possible relation to AF for SNPs within 1 Mb of the significant loci from the datasets, and incorporating SNPs that portrayed a minor allele frequency greater than or equal to 0.05. For the European and Japanese cases, 49 and 500 SNPs were selected, respectively. These selected SNPs underwent genotyping to be further analyzed in the replication study. Only the Japanese cases underwent a second round of replication study in which six SNPs were selected to be analyzed [18, 38–40].

After the validation studies, SNPs that exceeded or came close to genome-wide significance were searched for in the Cleveland Clinic Atrial Tissue Bank and the GTEx Portal of the Broad Institute of Harvard and MIT for eQTL analyses. This was done to understand variations in gene expression of the genotyped variants which led to the determination of six candidate genes. To determine the correlation of these candidate genes from the eQTL with the five novel genetic loci discovered in this study and nine genetic loci for AF discovered in previous studies [18, 38–40], gene enrichment analyses were performed. They showed the function relationship among those groups of genes. Genetic variants that cause gene expression changes can result in morphological and functional alterations in the cardiac muscle. This was tested using candidate gene knockdown in an embryonic zebrafish model. Co-immunoprecipitation of candidate genes was also done to determine the protein interaction of the novel candidate genes that were discovered in this study. Logistic regression models were applied to the European cohorts to calculate the association of the SNPs to AF. In the Japanese cases, the Cochran-Armitage trend test was used to exhibit the association of SNPs with AF; the most significant associations were further analyzed to validate the results.

Of the 49 SNPs tested in the European replication case profile of this study, four appeared to exceed genome-wide significance. After the two Japanese replication studies that comprised a total of 7530 AF cases, two SNPs had exceeded genome-wide significance in relation to AF. The results obtained included five new loci for AF (one of which was found to be associated with both Japanese and Europeans): chromosome 10q24 in both Japanese and Europeans, chromosome 12q24 in Europeans, chromosome 3p25 in Europeans, chromosome 6q22 in Europeans, and chromosome 12q24 in Japanese individuals. The gene related to the most significant variant on chromosome 10q24 was *NEURL* associated with SNP rs1241550 in Europeans and rs6584555 in Japanese. *NEURL* encodes an E3 ubiquitin ligase [45] which can suppress malignant astrocytic tumors [46]. The gene related to chromosome 12q24 was *TBX5* and SNP rs10507248 in Europeans. *TBX5*, a transcription factor, is a prominent gene in creating the cardiac conduction system [47]. The gene related to chromosome 3p25 was gene *CAND2* and SNP rs4642101 in Europeans. *CAND2* is important for myogenesis, as its codes for the TATA-binding protein [48]. In striated muscle tissue, the SNP rs4642101 decreased *CAND2/TIP120b* expression. The gene related to chromosome 6q22 was a larger region that was not specifically identified, and the SNP was rs13216675 in Europeans. The closest gene to this region is *GJA1*, which encodes connexin43, the gap junction protein that is largely expressed throughout the heart muscle [49]. More specifically, the left atrium and the whole heart were affected by the impact of the SNP on the transcription of *GJA1.* Finally, related to chromosome 12q24 was the gene *CUX2* and SNP rs6490029 in Japanese individuals.

To further investigate the role of these genes in cardiovascular genes, zebrafish knockdown studies of *NEURL*, *CAND2*, and *CUX2* were performed. *TBX5* and *GJA1* were not included in the knockdown studies as they are already well established in terms of protein function. Out of the specific categories measured—the validity and structure of the results of gene knockdown, impact on resting heart rate, contractility of the ventricles, and atrial action potential duration—only the atrial action potential duration showed distinct differences in the zebrafish knockdown studies [36]. The atrial action potential duration was increased in both knockdowns of the zebrafish *NEURL* and *CAND2* genes. Also, co-immunoprecipitation of *NEURL* revealed protein interaction with *PITX2* which resides near transcription factors. The eQTL loci mapping investigated the relationship between the novel loci and gene expression. The SNP rs4642101 resulted in the upregulation of *CAND2* expression in skeletal muscle. In left atrial samples, eQTL analyses revealed that the AF risk allele, SNP rs13216675, downregulated the expression of the gene *GJA1*, while the SNP rs10507248 upregulated the expression of the gene *TBX5*. There were also other genes related to AF in European Caucasians among the 49 SNPs initially analyzed; however, their association with AF was limited even though they met the significance threshold. Specifically, the expression of gene *CEP68* was correlated with SNP rs2723065 and SNP rs2540950. The other SNPs rs12733930, rs6490029, and rs2358891 were found to be in correlation with *LINC00467*, *TMEM116*, and *WIPF1*, respectively. There was also some correlation to the gene *NKX2.5*. Testing for implicated loci pathways, by ingenuity pathway analysis, identified that the most prominently enriched pathways were those linked to calcium signaling, L-serine degradation, and geranylgeranyl diphosphate biosynthesis. As AF is closely related to the risk of stroke, the five novel genetic loci discovered in this study were phenotypically analyzed to be in correspondence with ischemic stroke in the METASTROKE collaboration. The SNPs rs13216675 and rs10507248 in relation to genes *GJA1* and *TBX5*, respectively, demonstrated a correlation to cardioembolic stroke, along with the other three genes *NEURL* (rs12415501), *CAND2* (rs4642101), and *CUX2* (rs6490029).

As the study focused on European and Japanese subjects, it is limited in the application to other races. Also, the AF-associated SNPs are a marker for the risk of disease rather than the causal variants, a limitation for many GWAS studies. The scientists also believe their eQTL, co-immunoprecipitation, and zebrafish studies were crucial in their analysis, but to fully determine how these genes contribute to the pathogenesis of AF, further fine mapping, sequencing, and functional studies are needed. The five new loci identified, expand the range of genetic pathways that link to AF.

### Association of rare genetic variants and early-onset AF in ethnic minority individuals [50]

In recent years, progress has been made in understanding the global DNA architecture of AF; however, racial and ethnic populations have largely been underrepresented in these studies. The study by Chalazan et al. [50] aimed to fill this gap by outlining rare and novel pathogenic genetic variants linked to AF in ethnic minority individuals. All subjects in the family-based cohort were of African and Hispanic descent, age 18 and above, and taken from the University of Illinois at Chicago and Jesse Brown Veterans Administration Medical Centers. The subjects presented with early-onset AF which is the development of AF at the age of 66 or below and went through sequencing for the 60 candidate genes. Patients with AF which was a result of cardiothoracic surgery were not included in the cohort. The cohort had a mean onset age of 51 and consisted of 132 male probands and 95 female probands with 148 being African-American and 79 being Hispanic. The clinical characteristics of the participants included, but were not limited to, hypertension, type II diabetes, congestive heart failure, coronary artery disease, valve heart disease, left ventricular ejection fraction, and vascular disease. All consented participants had their family history taken and a family pedigree drawn. Targeted sequencing was done for the 60 candidate genes, which have been known to allow the susceptibility of AF. Using a stepwise approach for filtering, Chalazan et al*.* determined the genetic variants, both novel and rare, among their sample population. To validate any variants as possible disease-causing linkages to AF, the criteria of the American College of Medical Genetics and Genomics, Association for Molecular Pathology, and Association for Clinical Genomic Science were met [51, 52]. They grouped the variants into the following categories, pathogenic, likely pathogenic, having an unknown significance, benign, or likely benign. Sanger sequencing was performed on the rare and novel genetic variants from the first-degree family members and probands to confirm their relation to AF.

Fifty-three variants were identified that may increase the susceptibility of AF. Twenty-three rare variants were classified as unknown significance because these disease-causing variants were not found in the probands used in the study. Thirty novel variants were also identified with 17 of them pathogenic or likely pathogenic, and the remaining were of unknown significance. Six of these 30 variants produce ion channels in the heart. More specifically, the *SCN5A* gene, with a cytogenetic location on chromosome 3p22.2, that encodes cardiac sodium channels [53] was associated with three of these six variants. Another one of these six variants was specific for *CACNA1C*, the L-type calcium channel gene [53] with a cytogenetic location on chromosome 12p13.33. The last two of the six ion channel variants were specific to *KCNQ1* and *KCNJ11*, the potassium channel genes [53] with a cytogenetic location on chromosome 11p15.5-p15.4 and 11p15.5, respectively. Variants on the *TTN* gene were found in eight probands. This gene encodes for a large sarcomeric protein known as titin, and variants on this gene are a determined cause of AF [54]. In addition, there were genes that had a total of three rare and three novel variants of unknown significance that co-segregated with AF. The genes with the rare variants included: gene *SCN10A* with a cytogenetic location on chromosome 3p22.2 that encodes the sodium-channel protein type 10 subunit alpha [55], gene *KCNE5* with a cytogenetic location on chromosome Xq23 that encodes the voltage-gated potassium channel accessory subunit five [56], and gene *TTN* that encodes for titin. The genes with the novel variants included *MYH6* and NPPA. The gene *MYH6,* with a cytogenetic location on chromosome 14q11.2 that encodes for myosin heavy chain 6, had two variants, whereas *NPPA,* which has a cytogenetic location on chromosome 1p36.22 that encodes for natriuretic peptide precursor A, had one variant [57].

A limitation of this study was the low power because of the small sample size used. The study sequenced 60 candidate genes and regardless of this extensive panel, other candidate genes are still unknown. There was also a lack of screening on an extended list of myocardial structural protein genes. As a result, there may be a lower estimate than appropriate for the pathogenic variants that may lead to AF in these ethnic minorities. Chalazan et al*.* also stated they may have missed a limited number of variants found in the exonic regions.

### Variants in ***ZFHX3*** are associated with AF in individuals of European ancestry [37]

Prior to this study, SNPs at the genetic locus on chromosome 4q25 were known to be associated with risk of AF. Authors of this study, Benjamin et al*.*, hypothesized that further genetic variation may be the underlying etiology of AF [37]. Using participants from five community-based cohorts, this meta-analysis of genome-wide association studies for AF identified a new locus: *ZFHX3*. In this study, the participants were from existing GWAS data from the Heart and Aging Research in Genomic Epidemiology (CHARGE) cohorts [58]. CHARGE included the following five community-based cohorts: Age, Gene/Environment Susceptibility Reykjavik Study (AGES); Atherosclerosis Risk in Communities (ARIC); Cardiovascular Health Study (CHS); Framingham Heart Study (FHS); and Rotterdam Study (RS) [58]. Upon DNA collection, the mean ages of participants for prevalent AF cases were 76.5 (AGES), 72.3 (CHS), 65.5 (FHS), and 69.4 (RS). Similarly, the mean ages of participants for incidental AF cases ranged from the following: 76.3 (AGES), 57.0 (ARIC), 72.2 (CHS), 64.7 (FHS), and 69.1 (RS). In total, there were 896 prevalent AF cases with 15,768 controls and 23,854 incidentals AF cases with 21,337 controls. The validation study was based on the German AF Network (AFNET) consisting of 2145 AF cases and 4073 controls. All the subjects were of European ancestry with a significant number of the participants also presenting with hypertension, while others had diabetes, myocardial infarction, and heart failure.

All the community-based cohorts from CHARGE underwent genotyping using various genotyping arrays with multiple calling algorithms: BeadStudio (AGES), Birdseed (ARIC), BeadStudio (CHS), BRLMM (FHS), and BeadStudio (RS). The minor allele frequency for all the cohorts was less than 0.01, except the CHS cohort which did not drop SNPs with no heterozygotes. The imputation software for all the cohorts was as follows: Mach1 v1.0.16 (AGES and ARIC), BIMBAM v0.99 (CHS), and Mach1 v1.0.15 (FHS and RS). Each cohort demonstrated a different number of SNPs used for imputation, those being 308,340 (AGES), 602,642 (ARIC), 306,655 (CHS), 378,163 (FHS), and 530,683 (RS). The utilization of reference genotype data and linkage disequilibrium patterns were required to accomplish this.

The SNP rs2106261, intronic to the gene *ZFHX3*, a transcription factor with cardiac expression [37], was found to be another genetic locus for AF. The function of *ZFHX3* in cardiac tissue was unclear, but it was found to be expressed in mouse hearts [59]. Located on chromosome 16q22, *ZFHX3* is associated with the genetic roles of regulating myogenic [60] and neuronal differentiation [61]. *ZFHX3* has the genetic ability to suppress several cancers [62] and has been identified to play a role in the likelihood of developing Kawasaki disease [63]. To further support these results, a validation study using a cohort was conducted with the German AFNET. Analyses of both prevalent and incident AF cases in the discovery study involving the five community-based cohorts from CHARGE and the validation study supported that the SNP rs2106261 has a prominent relation to AF. Also, it was stated that there is a possibility that on chromosome 1p36, the gene *MTHFR* may be related to AF as it is in linkage disequilibrium (LD) with *NPPA* [37]. The *MTHFR* gene encodes for 5,10-methylenetetrahydrofolate reductase, while the *NPPA* gene encodes natriuretic peptides in the atrium [57]. It was described that familial AF was a resultant event of a frameshift mutation in *NPPA.* However, this notion merits further research as this was not confirmed by the validation study conducted with the AFNET cohort. It should also be noted that the previously discovered genetic locus for AF, SNP rs17042171 on chromosome 4q25 in relation to the gene *PITX2*, was replicated in this study as well.

Benjamin et al*.* noticed heterogeneity of effect sizes between studies. This could have occurred due to a few reasons which included but were not limited to, haplotype structure variations, AF length and causative factors, and differences in traits of cohort members. Another limitation of this article is that its basis of discovery is only for those of European ancestry and cannot necessarily be applied to other races. For the previously validated *PITX2* locus, heterogeneity within the study was observed which should be further studied. Also, this study focused on single SNPs meaning they neglected to take the patterns of haplotypes into consideration, eliminating complex haplotype associations from the results. Instead of being functional variants, the genetic variants found to be associated with AF in this study may be in linkage disequilibrium with causal variants.

### Meta-analysis identifies new susceptibility loci for AF [38]

This GWAS meta-analysis by Ellinor et al*.* [38] identified six novel AF loci, which are related to genes involved in cardiac ion channels, cellular signal transduction, and cardiopulmonary development. The results were reproduced in a replication study to support the findings. Four of the six novel loci were then investigated again as they played a role in AF in individuals of Japanese descent. The discovery study included subjects of European descent containing 6707 subjects with AF and 52,426 without AF. The following cohorts provided these individuals: The Age, Gene/Environment Susceptibility Study (AGES) Reykjavik study [64], the Atherosclerosis Risk in Communities (ARIC) study [65], The Cleveland Clinic Lone AF GeneBank Study [36], the Cardiovascular Health Study (CHS) [66], the Framingham Heart Study (FHS) [67], the German Competence Network for Atrial Fibrillation (AFNET/ KORA) [68, 69], The Massachusetts General Hospital AF Study (MGH) [36], The Heart and Vascular Health Study (HVH) [40], The Rotterdam Study (RS-I) [68], the Study of Health in Pomerania (SHIP) [69], The Vanderbilt Lone AF Registry, and The Women’s Genome Health Study (WGHS) [70]. The ages of the cohorts were 45–64 years during enrollment (AGES), 18 years or older (Cleveland Clinic Lone AF GeneBank Study), 65 years or older (CHS), not applicable (FHS), AF onset before 60 years (AFNET), less than 66 years (MGH), less than 66 years (HVH), less than or equal to 55 years (RS-I), 20–79 years (SHIP), 18–65 years (Vanderbilt Lone AF Registry), and not applicable (WGHS).

The replication study conducted to support the findings from the original GWAS included 5381 subjects with AF and 10,030 subjects without AF, all European ancestry. Subjects with AF were from the AFNET study, and the Cooperative Health Research in the Region Augsburg (KORA) provided the control subjects without AF. Other subjects with AF, at the age of 66 or greater, and the controls were used from the HVH study for replication. More AF cases and controls were acquired from MGH AF Study and the hospital catchment, respectively. From the Health Aging and Body Composition (Health ABC) study, African and Caucasian individuals were randomly sampled from ages 70–79. The Malmö Study, which consisted of prevalent and incident AF cases from Malmö, Sweden, The Ottawa Heart AF study, which consisted of lone AF or AF and hypertension, and the Ottawa Heart Genomics Study, which consisted of control groups (males being aged 65 or greater and the females being aged 70 or greater), were also among the validation cohorts. A final in silico GWAS study was performed to test the generalizability of the original study; four of the original six discovered loci were related to AF in Japanese individuals. This validation study included 843 individuals with AF and 3350 individuals without AF from the Japan BioBank project. Overall, the participants varied in age, and some displayed the following pre-existing conditions: hypertension, diabetes, heart failure, and myocardial infarction.

In the discovery stage of this study, all participants of the GWAS had their AF status assessed, and genotyping was performed. The number of SNPs with a minor allele frequency greater than 0.005 used in the study were 2,521,723 (AFNET/ KORA), 2,408,991 (AGES), 2,512,759 (ARIC), 2,319,581 incidental AF SNPs and 2,317,847 prevalent AF SNPs (CHS), 2,509,367 (CCAF), 2,501,666 incidental AF SNPs and 2,501,188 prevalent AF SNPs (FHS), 2,316,203 (HVH), 2,508,401 (MGH), 2,502,002 incidental AP SNPs and 2,501,903 prevalent AF SNPs (RS-I), 2,598,639 (SHIP), 2,543,887 (Vanderbilt), and 2,608,508 (WGHS). In each cohort, logistic or proportional hazards regression models were created as the primary statistical analysis. Adjustments were made for the age of participants when their DNA was drawn, their sex, and for the study site in cohorts ARIC and CHS. After quality control and meta-analyses of SNPs, a culmination of 2,609,549 SNPs from the cohorts were included in the study. SNP functions were predicated through the SNAP Proxy Search that obtained proxies for three previously known AF SNPs [37, 39, 40] and seven top SNPs identified in this study (one of which did not replicate in the replication cohorts). They were referenced against the HapMap (release 22) CEU population reference panel. To perform eQTL analyses, the GTEx eQTL browser was utilized and the dbSNP database allowed for the determination of functional annotations for the SNPs.

This extensive study revealed six new loci for AF. The first and most prominent genetic loci associated with AF were related to gene *PRRX1* on chromosome 1q24 (SNP rs3903239). *PRRX1* encodes a homeodomain transcription factor that is highly expressed in the connective tissue of the heart as it is developing [71]. The second locus was located on gene *CAV1* on chromosome 7q31 (SNP rs3807989). *CAV1* is expressed in the atria and encodes caveolin-1, a cellular signal transduction protein [72]. The protein encoded by the *CAV1* gene represses *KCNH2* expression [73], a gene previously found to be in relation to AF. The third locus was present on gene *SYNE2* on chromosome 14q23 (SNP rs1152591). *SYNE2* encodes nesprin-2 isoform proteins that have high expression in cardiac and skeletal muscles [74]. The fourth locus was on genes *FBP1* and *FBP2* on chromosome 9q22 (SNP rs10821415). These genes play vital roles in gluconeogenesis [75]. The fifth locus was found on gene *HCN4* on chromosome 15q24 (SNP rs7164883). The protein encoded by *HCN4* is highly expressed in the sinoatrial nodes of the heart as it is the main cardiac hyperpolarization-activated cyclic nucleotide-gated channel [76]. The sixth locus was found upstream of both genes *SYNPO2L* and *MYOZ1* on chromosome 10q22 (SNP rs10824026) whose proteins are expressed in cardiac and skeletal muscles. They also interact with various Z-disk proteins as well [77]. All these results were validated using cohorts, except a previously identified seventh locus which was related to gene *WNT8A* (SNP rs2040862). Next, these results were tested on individuals of Japanese descent in another GWAS. The genetic locus on gene *PITX2* on chromosome 4q25 (SNP rs2634073) surpassed the measures for genome-wide significance. Also, associated with AF in Japanese individuals was the genetic locus on chromosome 16q22 on gene *ZFHX3* (SNP rs12932445). On chromosome 1q21, the *KCNN3–PMVK* locus was not validated for the Japanese individuals at the same SNP as the Europeans. Instead, the SNP rs7514452 was found to be associated with AF in Japanese individuals rather than the SNP rs6666258 found in Europeans which is 375 kb away. The European SNPs in the genetic loci of genes *PRRX1* and *CAV1* on chromosomes 1q24 and 7q31, respectively, were also related to those of Japanese ancestry.

This study had several limitations. All consented individuals with AF, even those with some comorbidities, were included in the study to promote the highest level of generalizability of the results. These comorbidities included systolic dysfunction and hypertension. However, when referenced with the EchoGen Consortium, which contains electrocardiographic data from European individuals of various cohorts, none of the AF variants had meaningful correlations with systolic dysfunction. The datasets available in the GTEx eQTL browser were also a limitation to the eQTL analyses of the study.

### Identification of new genetic loci associated with AF in the Japanese population [78]

Low et al*.* conducted a study that led to the determination of genetic loci associated with AF in the Japanese population [78]. Six novel loci for AF were found in Japanese individuals. To perform the GWAS, a total of 8180 cases of AF, with 5713 of these cases being males, were used from BioBank Japan. 28,612 individuals were used as controls, with 11,223 of these cases being males, from the Tohoku Medical Megabank organization, the Japan Public Health Center-based Prospective study, and the Japan Multi-institutional Collaborative Cohort Study. The AF cases involved participants with ages ranging from 15 to 99, the mean age being 68.2 at the time of DNA collection, while the participants for control cases had ages ranging from 20 to 81, the mean being 56.2. Some of the participants had some pre-existing conditions that included diabetes, myocardial infarction, and heart failure, which the scientists confirmed did not change the results of the GWAS as they adjusted for these confounders and then determined any genetic association to AF. A validation study was also conducted to further support the newly found genetic loci which included 3120 AF cases, with 2383 men, from Tokyo Medical Dental University Hospital. Controls were a set of 125,064 sampled from BioBank Japan, 61,789 of these were males. The AF cases had ages from 18 to 93, the mean being 64.13, while the control participants had ages from 1 to 102, the mean being 60.77.

The genotyping of 713,014 common SNP variants and 273,000 functional exonic markers provided enough coverage with logistic regression analysis which was enlarged by whole-genome imputation. It allowed the inferences for any genotypes that may have been missing from the initial SNP arrays. The 1000 Genomes Project Phase I integrated release version 3 provided the reference panel for imputation, specific for descendants of East Asia. Allele frequencies in the GWAS were compared to those of the reference panels; SNPs that were unaccounted for were those that had differences greater than 0.16. In the validation study, Multiplex Invader assays were used to genotype the AF cases, while BioBank Japan supplied the genotype for the controls. In the AFGen Consortium that included a meta-analysis of GWAS data for those of European descent (15,993 AF cases and 113,719 controls), an in silico validation study was also conducted to ensure the genetic loci determined were linked to Japanese individuals. Pathway analysis was performed to reveal AF-related genetic pathways based on the Gene Ontology, KEGG, PANTHER, Biocarta, and Reactome databases. Through the Gene Ontology database, it was found that AF was related to the developmental pathway of the neural crest. An eQTL analysis using GTEx data facilitated the understanding of whether genetic variants identified in the study impacted tissue expression. Weighted genetic risk scores were determined to calculate the total risk of AF from the accumulation of the effect of 15 SNPs that were deemed to be significant from this GWAS.

At first, this GWAS determined 16 genetic loci associated with AF, and seven of these had been reported in previous studies. The other nine genetic loci underwent genotyping and association studies through which six genetic loci were found to be successfully associated with AF susceptibility in the Japanese population. The SNPs and their related genes include SNP rs12044963 in *KCND3* on chromosome 1p13, SNP rs17461925 in *PPFIA4* on chromosome 1q32, SNP rs2540953 in *SLC1A4/CEP68* on chromosome 2p14, SNP rs7698692 in *HAND2* on chromosome 4q34, SNP rs2296610 in *NEBL* on chromosome 10q12, and SNP rs2047036 in *SH3PXD2A* on chromosome 10q24. The most significant genetic locus was SNP rs7698692 in *HAND2* on chromosome 4q34. The *HAND2* gene plays a role in forming the right ventricle and aortic arch arteries as it encodes a cardiac morphogenesis protein [79, 80]. This SNP was observed to be prominent in those of Japanese descent since the minor allele of this variant is more prevalent in the East Asian population. The following significant genetic locus was SNP rs17461925 in *PPFIA4* on chromosome 1q32.1. The gene *PPFIA4* encodes a protein that binds to the leukocyte common antigen-related family receptor protein tyrosine phosphatases [81]. As a result, *PPFIA4* impacts axon guidance. The *SH3PXD2A* gene encodes a scaffolding protein that is necessary to produce the invadosome [82]. Inhibiting the *SH3PXD2A* gene from producing this protein affects the regulation of axon guidance. However, further studies are needed to determine the role of axon guidance in AF. The *KCND3* gene encodes a protein that has many functions related to AF pathogenesis. These include heart rate and smooth muscle contraction, neural excitability, and secretion of neurotransmitters. The risk allele of this SNP is more commonly seen in the East Asian population. Early onset AF was found to be in correlation to a gain of function mutation in *KCND3* [83]. The gene *NEBL* encodes a nebulin-like protein that has a role in myofibril assembly in the heart [84]. This protein binds to actin which interacts with the thin filaments and the other proteins associated with the Z-line, in striated muscle. As with previous variants, the risk allele of the SNP is more prevalent among the East Asian population as opposed to other populations. The eQTL analyses revealed how genes *PPFIA4* and *SLC1A4/CEP68* were affected by their respective SNPs. There was an upregulation of *PPFIA4* expression in whole blood and the aorta because of SNP rs17461925. The SNP rs2540953 downregulated *CEP68* expression in the tibial artery, cardiac atrial appendage, cardiac left ventricle, skeletal muscle, esophagus musculature, aorta, and subcutaneous adipose tissue. Similarly, in the tibial artery, the SNP rs2540953 changed the expression of *SLC1A4.* The clinical cardiovascular disease traits in relation to these AF genetic loci included, but were not limited to, ECG PR interval, ECG QRS duration, ischemic and cardioembolic stroke, and atrial flutter.

### Multi-ethnic GWAS for AF [85]

Roselli et al. [85] conducted a large, combined-ancestry meta-analysis of GWAS studies to identify 97 genetic loci associated with AF, of which 67 were novel. This study utilized approximately half a million participants from 50 studies. A total of 65,446 of these subjects were AF cases. As this study was a combined-ancestry meta-analysis GWAS, the ethnic groups of all the individuals were broken down as follows: 84.2% European, 12.5% Japanese, 2% African-American, and 1.3% Brazilian and Hispanic populations. Most of the individuals in the study were from the Broad AF study and AF Genetics consortium. The AF Genetics consortium contributed 23,685 AF cases and 148,192 controls, the Broad AF study contributed 17,517 AF cases and 10,987 controls, the UK BioBank contributed 10,064 AF cases and 334,953 controls, and the BioBank Japan contributed 8180 AF cases and 28,162 controls. The study utilized most individuals of European ancestry which included 55,114 AF cases and 482,295 controls. The number of participants of the other ethnic groups included 1307 AF cases and 7660 controls of African-American ancestry, 8180 AF cases and 28,612 controls of Japanese ancestry, 568 AF cases and 1096 controls from Brazil, and 277 AF cases and 3081 controls of Hispanic ethnicity. Some of the male and female participants, of various ages, presented with pre-existing conditions that included hypertension, diabetes mellitus, myocardial infarction, and heart failure.

The studies were mainly taken from the AF Genetics (AFGen) consortium and the Broad AF study. The UK Biobank (UKBB) and BioBank Japan (BBJ) were sources of additional results for the study. The Haplotype Reference Consortium (HRC) reference v1.1 panel on the Michigan Imputation Server v1.0.1 was used to perform imputation on studies with European and Brazilian ancestry, while studies of Japanese and Hispanic ancestry had imputation performed to the 1000 Genomes Phase 1 integrated v3 panel. The African-American ancestry studies used either method for imputation. Analysis of 8,328,530 common variants and 936,779 rare variants was performed, and the majority of the samples’ variants from the SNP array data were imputed with the HRC reference panel. Gene pathway enrichment by MAGENTA showed 55 pathways. To determine whether these AF genes associated with the genetic loci identified from the GWAS, RNA sequencing, and eQTL analyses were performed on 101 samples from the left atrium. Also, with the use of the GTEx project, eQTLs were found in the right atrial and left ventricular tissue samples of the heart. The MetaXcan method was used to perform a transcriptome-wide analysis to draw conclusions about the correlation between disease risk and genetically predicted gene expression. Fifty-five genes associated with AF were found using transcriptome-wide analysis of which 42 of these 55 genes correlated with AF genetic loci found in the GWAS. A phenome-wide association study (PheWAS) was also performed to associate 12 risk factors with AF using the UK Biobank.

The corresponding SNPs and genes to the 67 novel genetic loci are further described based on chromosome number (Table 1). On chromosome 1, there were six genetic loci identified whose nearest genes were *UBE4B, CASZ1, C1orf185, CASQ2, GJA5,* and *NUCKS1*. On chromosome 2, there were five genetic loci identified whose nearest genes were *KIF3C, XPO1, REEP1/KDM3A, WIPF1/CHRNA1,* and *SPATS2L*. On chromosome 3, there were three genetic loci identified whose nearest genes were *LRIG1, PHLDB2,* and *GNB4*. On chromosome 4, there were four genetic loci identified whose nearest genes were *WDR1, SLC9B1, CAMK2D,* and *ARHGAP10*. On chromosome 5, there were two genetic loci identified whose nearest genes were *ARHGAP26/NR3C1* and *NKX2–5*. On chromosome 6, there were four genetic loci identified whose nearest genes were *ATXN1, KDM1B, CDKN1A,* and *UST*. On chromosome 7, there were five genetic loci identified whose nearest genes include *DGKB, CREB5, GTF2I, COG5,* and *KCNH2*. On chromosome 8, there were four genetic loci identified whose nearest genes were *XPO7, FBXO32, MTSS1/LINC00964,* and *PTK2*. On chromosome 9, there were three genetic loci identified whose nearest genes were *SLC24A2/MLLT3, ZNF462,* and *PSMB7*. On chromosome 10, there were three genetic loci identified whose nearest genes were *REEP3, C10orf11,* and *C10orf76*. On chromosome 11, there were two genetic loci identified whose nearest genes were *NAV2* and *SORL1/MIR100HG*. On chromosome 12, there were seven genetic loci identified whose nearest genes were *SSPN, PKP2, NACA, BEST3, KRR1/PHLDA1, TBX5-AS1/TBX3,* and *DNAH10*. On chromosome 13, there was one genetic locus identified whose nearest genes were *LINC00540/BASP1P1*. On chromosome 14, there were four genetic loci identified whose nearest genes were *MYH7, AKAP6, SNX6/CFL2,* and *LRRC74/IRF2BPL*. On chromosome 15, there were four genetic loci identified whose nearest genes were *USP3, TLE3/UACA, LINC00927/ARNT2,* and *IGF1R*. On chromosome 16, there was one genetic locus identified whose nearest gene was *RPS2*. On chromosome 17, there were four genetic loci identified whose nearest genes were *POLR2A/TNFSF12, MYOCD, MAPT,* and *KCNJ2/CASC17*. On chromosome 18, there was one genetic locus identified whose nearest gene was *SMAD7*. On chromosome 20, there were two genetic loci identified whose nearest genes include *CASC20/BMP2* and *C20orf166*. On chromosome 21, there was one genetic locus identified whose nearest gene was *LOC100506385*. On chromosome 22, there was one genetic locus identified whose nearest gene was *TUBA8*. An ancestry-specific meta-analysis for those of European ancestry also found three additional genetic loci in correlation to AF. They were in or near genes *CDK6, EPHA3,* and *GOSR2*. Upstream from the gene *PITX2*, on chromosome 4q25, is the region that is most prominently associated with AF in individuals of European, Japanese, and African-American ancestry.

Thirteen of the AF genetic loci were found to be missense variants. One example of the missense variants is SNP rs11057401 on gene *CCDC92* which was correlated with coronary artery disease [86]. Four out of the five in silico prediction algorithms determined this missense variant to be damaging. According to Roselli et al*.*, four significant themes were observed in the study. First, the following two genes associated with AF genetic loci are targets of AF medication. The *SCN5A* and *KCNH2* genes encode a cardiac sodium ion channel and alpha subunits in the cardiac potassium channel complex, respectively [48]. Sodium-channel blockers target the *SCN5A* gene [87] and potassium-channel-inhibiting medications target the *KCNH2* gene. Next, it was found that transcription regions have a significant causal relation to AF. The transcription factor genes, *TBX3* and *TBX5,* influence the cardiac conduction system development [88]. The transcription factor encoded by the *NKX2-5* gene signals the development of the heart. In relation to AF, this transcription factor has been linked to heart rate and congenital heart disease [89]. The *PITX2* gene encodes a transcription factor that when downregulated has an association with AF; it modifies both the action potential of the atria and the therapeutic efficacy of sodium-channel blockers [90]. Another theme, observed by the transcriptome-wide analyses, was the downregulation of *PRRX1* expression and the upregulation of *TBX5* and *KCNJ5* expression. The regulation of these genes in this manner has been correlated to AF. In particular, the IKACh current channel is upregulated in AF which is associated with a cardiac potassium channel encoded by the KCNJ5 gene. Finally, the Mendelian form of this arrhythmia was highlighted by the genes involved at the discovered AF loci. Cardiomyocyte communication and structure are diminished by variants in the *PKP2* gene. This disruption is common in arrhythmogenic right ventricular cardiomyopathy [91]. Another example is mutations in the *CASQ2* gene allow for the development of catecholaminergic polymorphic ventricular tachycardia [92]. Mutations in various genes, such as *GJA5, KCNH2, SCN5A, KCNJ2, MYH7,* and *NKX2-5* have a linkage to several diseases that include inherited arrhythmias, cardiomyopathy, and conduction system diseases in the heart [93].

Regarding the pleiotropic effects of the genetic loci, the SNP rs880315 at gene *CASZ1* produced the phenotypes of hypertension and blood pressure. With the Bonferroni correction, numerous SNPs of AF exhibited a significant association with twelve different phenotypes that included height, body mass index, smoking, hypertension, heart failure, stroke, bradyarrhythmia, mitral regurgitation, peripheral vascular disease, hypercholesterolemia, coronary artery disease, and type II diabetes. Other disease traits in relation to the AF genetic variants included, but were not limited to, HDL cholesterol, platelet count and aggregation, ECG QRS complex and duration, ECG QT interval, and heart rate. It was found that 64 out of the 67 novel loci identified in this study, resided in regulatory elements in cardiac tissue. The classified AF genetic loci implicated genes that were associated with cardiac electrophysical, structural, contractile, and developmental pathways. The 55 gene sets and pathways determined from the gene set enrichment analysis played a role in cardiac electrophysiology, cardiomyocyte structural and contractile groups, and cardiac development. Out of the 67 novel genetic loci determined by the study, 48 of the loci were related to gene(s) that were within 500 kb of the variants that were part of one of these pathways. The sample size for the eQTL analyses of the left atrium posed a limitation on being able to enhance the specificity of candidate genes for AF.

### Biobank-driven genomic discovery yields new insight into AF biology [94]

In this study, Nielsen et al. performed a GWAS to determine 111 genetic loci and 142 risk variants for AF [94]. 151 function candidate genes for AF were identified as well. Through pathway and functional enrichment analyses, it was found that many genes related to AF act by remodeling the cardiac structure, like atrial cardiomyopathy. This large GWAS was composed of approximately 1,000,000 participants that included 60,620 AF cases and 970,216 controls. The participants were accessed from six different studies: The Nord-Trøndelag Health Study (HUNT) [95], deCODE [96], the Michigan Genomics Initiative (MGI) [97], DiscovEHR [98], UK Biobank [23], and the AFGen Consortium [99]. All cases and controls were of European ancestry and at an age of 20 years and older. HUNT contributed 6493 AF cases and 63,142 controls gathered from an array of diagnosed discharges in the hospital, out-patient, and emergency rooms. Two of the largest hospitals in Iceland provided 13,471 AF cases and 358,161 controls, which were obtained from deCode. Located in Michigan Medicine, USA, the MGI cohort provided 1226 AF cases, while controls were those with no phenotypes of AF. 6679 AF cases and 41,803 controls were acquired from the DiscovEHR cohort, and 17,931 AF cases and 115,142 controls were obtained from the AFGen Consortium, while the remaining AF cases and controls came from the UK BioBank.

All the cohorts underwent genotyping and sequencing. WGS was done on 2201 individuals after which the GotCloud pipeline was utilized to produce genotype calls. A total of 15,220 Icelanders from the deCODE studies had whole-genome sequencing performed with the Illumina standard TruSeq methodology. During sequencing, the Genome Analysis Toolkit version 3.4.0 found autosomal SNPs and insertions or deletions of nucleotide bases in the samples. Logistic regression enabled the determination of associations between SNPs and AF. In the MGI cohort, genotyping was done using the Illumina Human Core Exome v1.0 and v1.1 at the University of Michigan and the Firth bias-corrected logistic likelihood ratio test was used to find the association between variants and AF. The DiscovEHR study had DNA aliquots genotyped on the Human OmniExpress Exome Beadchip. An additive genetic model was used to ascertain how the variants related to AF. The UK BioBank employed the Applied Biosystems UK BiLEVE Axiom Array and UK BioBank Axiom Array to genotype the study subjects. For the UK BioBank participants, a generalized mix model helped find the associations between the variants and AF. To provide an estimation of heritability, the GWAS summary statistics, linkage disequilibrium (LD) score regression, and European ancestry LD were analyzed. Nielsen et al. further investigated AF-causing genes, cell types and tissue which have AF-related gene expression, and to discover enriched gene sets for genes at the AF genetic loci. A total of 889 gene sets were detected to be enriched which are related to the structural remodeling of the cardiac muscle and cardiac development and morphology. Most of the genetic variants for AF were found to be in non-coding regions of the genome, so an algorithm called the ‘Genomic Regulatory Elements and GWAS Overlap andomiza’ (GREGOR) was implemented to identify the function of the non-coding variants related to AF. 3048 genes contained transcribed regions that were in conjunction with at least 1 variant from the 111 genetic loci identified in this study. One hundred and fifty-one functional candidate genes were prioritized from this, and ‘Tissue-Specific Expression Analysis’ (TSEA) was performed to reveal tissues in which the expression of these genes was intensified. ECG data were also examined from the Landspitali University Hospital located in Reykjavik, to perform ECG-wide association analyses. The relationship between the 123 ECG parameters and the 111 genetic variants determined from this study was recognized.

This GWAS identified 111 genetic loci for AF, of which 80 had not been previously reported. The corresponding SNPs and genes to these eighty novel genetic loci are further described based on chromosome number (Table 1). On chromosome 1, there were seven genetic loci identified whose nearest genes include *CASZ1, HSPG2, SCMH1, AGBL4, CASQ2, GJA5,* and *NUCKS1/SLC41A1*. On chromosome 2, there were eight genetic loci identified whose nearest genes include *KIF3C, USP34, REEP1, GYPC, TEX41, WIPF1, SPATS2L,* and *ERBB4*. On chromosome 3, there were eight genetic loci identified whose nearest genes include *THRB, LRIG1/SLC25A26, FRMD4B, EPHA3, PHLDB2/PLCXD2, PPP2R3A, GNB4,* and *XXYLT1*. On chromosome 4, there were four genetic loci identified whose nearest genes include *FGF5, SLC9B1, CAMK2D,* and *ARHGAP10*. On chromosome 5, there were five genetic loci identified whose nearest genes include *LOC102467213, SLC27A6, NR3C1, SLIT3,* and *NKX2-5*. On chromosome 6, there were five genetic loci identified whose nearest genes include *ATXN1, KDM1B/DEK, CDKN1A/PANDAR/PI16, CGA,* and *UST*. On chromosome 7, there were six genetic loci identified whose nearest genes include *DGKB, CREB5, GTF2I/LOC101926943/GTF2IRD2, CDK6, OPN1SW/CALU,* and *KCNH2*. On chromosome 8, there were four genetic loci identified whose nearest genes include *GATA4, XPO7, FBXO32,* and *PTK2*. On chromosome 9, there was one genetic locus identified whose nearest gene was *LHX3*. On chromosome 10, there were four genetic loci identified whose nearest genes include *REEP3/NRBF2, SIRT1/MYPN, C10orf11,* and *RBM20*. On chromosome 11, there were two genetic loci identified whose nearest genes include *NAV2* and *SORL1*. On chromosome 12, there were seven genetic loci identified whose nearest genes include *SSPN, PKP2, NACA, LRRC10, PHLDA1, HIP1R,* and *FBRSL1*. On chromosome 13, there were two genetic loci identified whose nearest genes were *LINC00540/LINC00621/SGCG* and *CUL4A*. On chromosome 14, there were four genetic loci identified whose nearest genes include *AKAP6, CFL2, DPF3,* and *IRF2BPL*. On chromosome 15, there were three genetic loci identified whose nearest genes include *HERC1, ARNT2, and IGF1R*. On chromosome 17, there were five genetic loci identified whose nearest genes include *YWHAE/CRK/MYO1C, TNFSF12/TNFSF12–TNFSF13/SOX15/FXR2, MYOCD, ZPBP2/GSDMB/ORMDL3,* and *CYTH1/USP36*. On chromosome 18, there were two genetic loci identified whose nearest genes were *SMAD7* and *MEX3C*. On chromosome 21, there was one genetic locus identified whose nearest gene was *LINC01426*. On chromosome 22, there were two genetic loci identified whose nearest genes were *TUBA8* and *MYO18B*.

To further specify, a minimum of 18 genes were found to be involved in cardiac and skeletal muscle. These genes include *AKAP6, CFL2, MYH6, MYH7, MYO18B, MYO1C, MYOCD, MYOT, MYOZ1, MYPN, PKP2, RBM20, SGCA, SSPN, SYNPO2L, TTN, TTN-AS,* and *WIPF1*. Two genes, *RBM20* and *PKP2*, have been associated with more specific cardiac conditions, dilated cardiomyopathy [100] and arrhythmogenic right ventricular cardiomyopathy [101], respectively, while the gene *SCGC* is associated with the skeletal muscle condition, muscular dystrophy [102]. The 13 genes that were also established to likely intervene in developmental events include *ARNT2, EPHA3, FGF5, GATA4, GTF2I, HAND2, LRRC10, NAV2, NKX2-5, PITX2, SLIT3, SOX15,* and *TBX5* [103]. The genes *CALU, CAMK2D, CASQ2,* and *PLN* play a role in calcium handling in cardiac cells [104–106], and the genes *TNFSF12* and *TNFSF13* are associated with angiogenesis. Furthermore, the genes *CGA, ESR2, IGF1R, NR3C1,* and *THRB* are linked to hormone signaling, and the genes *HCN4, KCND3, KCNH2, KCNJ5, KCNN2, KCNN3, SCN10A, SCN5A,* and *SLC9B1* are related to cardiac ion channels [107]. The functional candidate genes *MYH6* and *MYH7* are connected to index variant rs422068 located on chromosome 14. The role of these genes is to encode proteins for molecular motors of the myocardium [108, 109]. ATP hydrolysis produces chemical energy which these molecular motors convert into mechanical energy. Once completed, mechanical energy can be used to support the heartbeat. In cardiac conditions, including heart failure, the α-myosin heavy chain produced by the MYH6 gene is downregulated and the β-myosin heavy chain produced by the *MYH7* gene is upregulated. *MYH6* and *MYH7* working in this manner reduce cardiac performance. In relation to AF, *MYH7* expression has been seen at elevated levels in patients’ atrial myocytes, in recent studies.

DEPICT revealed 889 gene sets that portrayed varying degrees of enrichment for the genes pertaining to AF loci. The gene sets highly enriched that pertain to genes of AF loci include those for thick ventricular wall, abnormal cardiovascular system physiology, enlarged myocardial fiber, enlarged heart, disorganized myocardium, complete embryonic lethality during organogenesis, decreased cardiac muscle activity, increased heart weight, abnormal cardiac muscle contractility, positive regulation of cell differentiation, atrial septal defects, and decreased embryo size. The gene sets enriched to a lower degree include those for abnormal heart morphology, muscle structure development, failure of heart looping, abnormal heart development, trabecula carnea hypoplasia, heart development, and KEGG arrhythmogenic right ventricular cardiomyopathy. The 111 AF loci found in his study were assessed for relation to various electrographic traits. After measuring their correlation with 123 electrocardiogram parameters, it was found that at a minimum, one electrocardiogram parameter was related to 60 AF loci. Thirty-nine of these 60 loci were novel. More specifically, these parameters encompassed but were not limited to, both PR and QT intervals, P-wave duration, and heart rate. The genes related to the variants with the most prominent effects on the measured ECG traits included: *NACA, THRB, CAMK2D, NKX2-5,* and *CDKN1A*.

## Studies examining genes associated with heart failure (HF)

The remainder of the review examines/focuses on the literature reporting various techniques and methods used to identify genes related to HF (Fig. 1).

### Genome-wide association and multi-omics analyses reveal ACTN2 as a gene linked to HF [4]

This clinical study by Arvanitis et al*.* involved GWAS analysis for HF using a multi-omics approach to ultimately detect and replicate a known locus on chromosome 4 near the *PITX* gene and two novel loci on chromosome 9 near the *ABO* gene and on chromosome 1 near the *ACTN2* gene which both correlated to HF [4]. The new locus on chromosome 1 near the *ACTN2* gene plays a role in HF and left ventricular remodeling. During cardiomyocyte differentiation, a cardiac muscle-specific regulatory region binds to the promoter of the *ACTN2* gene, which suppresses its expression. This leads to the elimination of the identified novel regulatory region. In this study, a large-scale GWAS meta-analysis of five cohorts was conducted with a sample population of European ancestry. It consisted of a total of 10,796 HF cases and 437,573 controls. Additionally, the study validated their results through a cohort of 24,829 self-reported HF cases and 1,614,513 controls of European ancestry from the company 23andMe.

The study conducted a GWAS in five cohorts Ih include the Framingham Heart Study, Cardiovascular Health Study, Atherosclerosis Risk in Communities Study, Multi-Ethnic Study of Atherosclerosis, Women’s Health Initiative, and the eMERGE cohort. Each one of the studies was imputed to the 1000 genomes phase three reference panel using Minimac23 along with pre-phasing with Eagle. A GWAS was conducted for HF with variables, including age, sex, and the first ten genotype principal components (PCs). The studies were validated using HF cases from a cohort and the company 23andMe. The PheWAS association of HF subtypes was concluded from the patient history of diseases or surgeries. Associations with other phenotypes were tested via the NHGRI-EBI GWAS catalog and GWAS atlas. The LD score regression was used to determine heritability and genetic correlation. Mendelian randomization (MR) analysis concluded the effects of AF on HF development from the GWAS. The variant fine mapping was approached in a stepwise manner based on epigenomic annotations and LD structure. ATAC-seq, ChIP-seq of H3K4me3 and H3K27ac, RNA-seq, and HiC experiments were performed in an engineered H9 hESC to create a cardiomyocyte differentiation model. The GTEx and the cis-eQTL summary statistics from eQTLGen were used to determine the effects of genome-wide significant variants in gene expression. A Bayesian colocalization method was utilized to identify the causal variant between eQTL and GWAS. The CRISPR–Cas9 system created a deletion in the causal region of the ACTN2 locus in hESCs, and the LeafCutter performed splice-QTL analysis on the ABO locus.

The LD score provided results to validate the genetic correlation between HF Ind its risk factors and found a significant correlation between HF and musculoskeletal traits. The chromosome 4 locus with the SNP rs1906615 resides near the *PITX2* gene; this locus is known to be correlated with AF, but in recent findings of the article, it also plays a role in HF [110]. MR analysis supported the correlation between AF and its role in HF development. Additionally, the MR Egger and weighted mean approach provided a value of statistical significance to further support the association. The chromosome 1 locus with the SNP rs580689 resides close to the *ACTN2* gene, and this gene is responsible for encoding structural cardiac protein in the sarcolemma region [111]. Additionally, it has been found that rare mutations in this area have led to the development of cardiomyopathy and, ultimately, HF. A PheWAS approach discovered the involvement of this locus in both ischemic and non-ischemic HF and left ventricular dilation. An experiment of ChiP-seq data of p300/CREBBp displaced the presence of cardiac muscle enhancer due to the peak in the identified region. The study confirmed that a regulatory region switches on during cardiomyocyte differentiation, and *ACTN2* is induced during maturation. On chromosome 9, SNP rs9411378 resides in the *ABO* gene, which is known for its role in regulating blood type and has been associated with the development of ischemic diseases [112]. Researchers wanted to explore whether the locus could affect the splicing of the *ABO* gene via RNA-seq data. The results showed that the locus was, in fact, associated with the splicing of *ABO* and promoted a splice variant that skips the exon on the SNP. The PheWAS approach produced results where the *ACTN2* locus was greatly correlated with both ischemic and non-ischemic HF and was found to be associated with left ventricular dilation and HF with reduced ejection fraction. The linkage between cardiovascular disease/cardiovascular mortality and having a non-O blood type through structural variation in the *ABO* gene is a correlation that has not been thoroughly explained. Yet, the study still highlights the significance of the ABO gene as well as its role in cardiovascular disease, although the mechanisms involved are left for future studies to discover.

### HF and sudden cardiac death in heritable thoracic aortic disease caused by pathogenic variants in the SMAD3 gene [113]

Heritable thoracic aortic disease (H-TAD) is a group of disorders correlated with aortic aneurysm or dissection in levels of the aorta which may be associated with aortic valve disease. The causal genes in the H-TAD group are sorted based on those that affect cardiac structure and those that can modify cardiac structures. The second group can further be divided into genes that take part in TGFB signaling and genes involved in vascular smooth muscle cell contractility. Additionally, evidence of variants in the FBN1 gene and its correlation to intrinsic myocardial dysfunction and Marfan syndrome (MFS) is significantly growing. Furthermore, prior cases of deaths in patients with MFS who dealt with HF and cardiac death have been documented and studied. This study by DeBacker and Braverman et al*.* looks at the SMAD3 variant, which can turn into forms of H-TAD, and its relation to three separate cases [113, 114].

A total of three cases were observed, with two cases coming from the same family. The first case was observed in an aging man from St. Louis (USA) who had a pre-existing condition of a bilateral inguinal hernia correction. He had an aortic root aneurysm of 50 mm. The family history indicated his father having an unconfirmed diagnosis of “MFS” and other members on his paternal side having aortic aneurysms and dissections. It was later disclosed that the patient had left ventricular dysfunction and underwent aortic root replacement. He eventually developed HF, and an implantable cardiac defibrillator (ICD) was fixed. Genetic tests reported the presence of the SMAD3 variant. The second case followed the history of a man from Ghent (Belgium) who had been diagnosed with idiopathic dilated cardiomyopathy at the age of 25. He had HF by the age of 27 and received surgery for an acute aortic dissection with the insertion of a left ventricular assist device (LVAD). He passed away shortly after. This patient had a family history of aortic dissection on his maternal side. In the same family, the daughter of the man from Ghent was found to display Marfanoid features, mild myopia, and mitral valve prolapse at the age of 5. She went under screening for the presence of the *FBN1* gene, and the results displayed a benign polymorphism. At the age of 21, the young woman’s echocardiography reports noted moderate dilation of the left ventricle along with no arrhythmias present. At 22, she experienced cardiac arrest and died shortly after.

All three cases possessed a variant of the *SMAD3* where two patients experienced end-stage HF, and one patient had lethal arrhythmias alongside moderate left ventricular dilation. The correlation of MFS with increased HF rates, myocardial dysfunction, and ventricular arrhythmias/cardiac deaths in patients has been established. Several additional studies have explored the idea that the absence of certain triggers could result in myocardial dysfunction. A smaller study concluded that left ventricular dilation in MFS patients is seen more in the non-missense *FBN1* pathogenic variant. There is an established phenotypic overlap between MFS and other H-TAD groups related to genes that are responsible for encoding parts for the TGFB signaling pathway, myocardial dysfunction, and arrhythmias. Since a small-scale study was conducted, a larger study with a greater sample size would need to be conducted to confirm these findings as well as further understand the mechanisms presented.

### Disruption of MAP7D1 gene function increases the risk of doxorubicin-induced cardiomyopathy and HF [115]

This clinical study by Li et al. observed the drug doxorubicin, which falls under the classification of medications called anthracyclines and serves as a chemotherapeutic drug used to treat different types of cancers. Researchers wanted to understand the relationship between threshold doses in patients and correlation to doxorubicin-induced cardiomyopathy (DIC) and HF [115]. Researchers aimed to find a link between the genetic susceptibility to DIC and HF due to the genetic variations in individual genomes from these diseases and accomplished this via a GWAS in humans and an animal model. The study utilized a zebrafish (*Danio rerio*) animal model due to its high-throughput genetic and compound screening as well as its frequent usage as an experimental model for human cardiac diseases. The BL6 WT mice were another animal model used to interpret cardiac function upon injection of doxorubicin (Sigma). The GWAS dataset consisted of 1191 breast cancer patients of different races: Asian, American, African, and European. The cancer patients had received adjuvant doxorubicin therapy alongside their participation in the n9831 phase III clinical trial.

In the zebrafish animal model, procedures were carried out as per the guidelines of the Institutional Guidelines for Animal Use and Care of Qingdao University and *Guide for the Care and Use of Laboratory Animals.* Genotyping PCR identified the GBT239/map7d1b mutant fish. The fish and mice were fasted for 24 hours before injection of doxorubicin, which was distributed based on body weight. Subsequent cardiac function and swimming capacity were measured four weeks after injection of the drug. Fasting was conducted 24 hours prior to the measurement of swimming capacity and measured using the swim tunnel respirometer. The Vevo 3100 high-frequency imaging system was utilized to perform echocardiography measurements. Heart tissues from animal models were homogenized in a RIPA buffer and western blot analysis was performed. The GWAS dataset of breast cancer patients had all received doxorubicin therapy from the N9831 phase III clinical trial. From the clinical trial, the *MAP7D1* variants associated with the degradation of cardiac function and HF were retrieved. A linear regression analysis was performed to determine the decline in left ventricular ejection fraction (LVEF).

Through the fluorescence reporting system in the gene-breaking transposon (GBT) system in zebrafish, cardiac gene disruption was identified. The GBT239 mutant was found to display cardiac and skeletal muscle-specific expressions and further investigation through the fluorescent reporting system displayed results of the mutant marked in the *MAPA7D1B* protein of the sarcomere in the adult zebrafish. In the doxorubicin-injected fish, there was a decline in ejection fraction (EF) and fractional shortening (ES), as well as myofibril loss and damage to the trabecular tissue. Swimming capacity was also significantly diminished in these mutant fish. Evaluation of *MAP7D1* gene function in human patients was noted by the marker of cardiomyocyte apoptosis, which is a crucial index for DIC in humans, by the transferase-mediated dUTP nick end labeling (TUNEL) assay. A high TUNEL index was also found in the mutant fish hearts upon investigation for cardiomyocyte apoptosis. Additionally, an impairment in autophagy flux was associated with DIC in the mouse model. From the GWAS dataset, a linear regression analysis was utilized and adjustments for age, baseline LVEF, antihypertensive, and medications were considered. As a result, two rare variants (rs272825 and rs272832) were discovered in the *MAP7D1* locus and are correlated to a decline in LVEF. These variants were identified after cancer patients received doxorubicin chemotherapy as part of their treatment routine. The two variants were correlated with a decline in LVEF with low P values and reports displayed that patients with one copy of the risk allele would have an average decline in LVEF of 27.24 points. Seventeen patients whose LVEF levels were monitored throughout the clinical trial experienced irreversible congestive heart failure (CHF), thus concluding that the decline in LVEF and CHF was correlated with the *MAP7D1* gene being a susceptible gene to DIC and HF. The gene increases the risk of DIC and HF in humans if mutations are found in the gene. Autophagy is a self-rejuvenation cellular process and plays a vital role in the maintenance of protein homeostasis. The *MAP7D1* gene is part of the microtubule-associated protein (MAP) family, and it plays the role of binding to microtubules and assisting in autophagosome formation [116]. Disruption in the microtubule dynamics could result in autophagy impairment and eventual protein aggregation, a crucial point found in elevated levels in the map7d1b mutant hearts. This concludes the role of the gene and its involvement in autophagy assembly and its relationship to DIC and HF.

In the clinical trial where the LVEF levels of the patients were monitored, a total of 17 patients were presented with complications of CHF, which is a small number in comparison to the GWAS analysis. For future studies, a definite gene list of genes linked to specific phenotypes such as DIC could provide researchers with more information and procure preventative measures for cancer patients who are at high risk of experiencing cardiomyopathy and HF after receiving doxorubicin chemotherapy.

### GWAS and Mendelian randomization analysis provide insights into the pathogenesis of HF [117]

Previous studies of monogenic hypertrophic and dilated cardiomyopathy (DCM) have indicated its correlation to HF. In this study by Shah et al., GWAS was performed to discover more genetic variants and find the correlation to risk factors [117]. The study had a sample size of 47,309 cases and 930,014 controls of European Ancestry comprising 26 studies from the Heart Failure Molecular Epidemiology for Therapeutic Targets (HERMES) Consortium. The sample included population cohorts (17 studies, 38,780 HF cases, 893,657 controls) and case–control samples (9 studies, 8529 cases, 36,357 controls). All the studies used genotyping, with pre-imputation quality control (QC) and imputation on reference panels. Study-level GWAS analysis was performed, and summary-level estimates were utilized for the meta-analysis. The meta-analysis was compiled using the IVW approach and inflation was tested using LDSC intercept. To identify variants that were associated with HF, researchers used FUMA. Heritability estimation was used to estimate genetic contribution. The 10,000 UK Biobank participants of European ancestry utilized the LD reference. MAGMA was used to perform a gene set enrichment analysis. Missense consequences of sentinel variants and proxies were queried using the Combined Annotation-Dependent Depletion (CADD) score. The GTEx v7 resource and MAGNet repository were used on two eQTL datasets to provide expression quantitative trait analysis. Bayesian colocalization was conducted to determine associations between gene expression and HF risk. Transcriptome-wide association analysis was performed on the GTExv7 heart tissue datasets. The INTERVAL study provided testing of protein quantitative trait analysis in blood. The association of HR risk loci with other phenotypes was performed using the NHGRI-EBI Catalog. Hierarchical agglomerative clustering was conducted using the complete linkage method. Genetic correlation analysis was estimated between HF and 11 risk factors using LDSC. Two-sample Mendelian randomization analysis was performed using the generalized summary data-based Mendelian randomization (GSMR). Conditional analysis was performed using the mtCOJO on summary data.

The study identified 12 variant associations for HF risk at genomic loci associated with HF that were of genome-wide significance. The genes and SNPs included: *CELSR2* with SNP rs660240 on chromosome 1, *PITX2* with SNP rs17042102 on chromosome 4, *FAM241A* with SNP rs17042102 on chromosome 4, *KLHL3* with SNP rs11745324 on chromosome 5, *CDKN1A* with SNP rs4135240 on chromosome 6, *LPA* with SNPs rs55730499 and rs140570886 on chromosome 6, *CDKN2B*-*AS1* with SNP rs1556516 on chromosome 9p21, *ABO* with SNP rs600038 on chromosome 9, *SURF1* with SNP rs600038 on chromosome 9, *SYNPO2L* with SNP rs4746140 on chromosome 10, *AGAP5* with SNP rs4746140 on chromosome 10, *BAG3* with SNP rs17617337 on chromosome 10, *ATXN2* with SNP rs4766578 on chromosome 12, and *FTO* with SNP rs56094641 on chromosome 16. Ten of the loci were not previously reported. The variants showed associations with one or more traits, including coronary artery disease (CAD), AF, body mass index (BMI), and reduced LV function. CAD gene *LPA*, AF gene *PITX2*, and BMI gene *FTO* were among the few that showed the strongest association with risk factors [118, 119]. Six variants were linked to CAD, 9p21/CDKN2B-AS1 and LPA, which were previously known, and four variants were linked to AF. In total, four loci were HF loci which were not associated with the HF risk factor, CAD. These include *KLHL3* and *SYNPO2L* (linked to AF) and the loci BAG*3* and *CDKN1* (correlated with LV systolic function). The BAG3 locus and correlation to DCM were also noted [120]. Through tissue-enrichment analysis, 13 genes were correlated with HF, and four of them were near an HF variant that was seen in heart tissue. In the eQTL dataset, eight of the 12 variants were correlated with cis-gene expression. The variant in the *SYNPO2l/AGAP5* locus expressed *MYOZ1* and *SYNPO2L* and had the role of binding Z-disk cardiac proteins. *MYOZ1* is a negative regulator of calcineurin signaling [121] and *SYNPO2L* is involved in cardiac development and sarcomere maintenance [122]. Researchers aimed to find some of the risk factors and found that higher systolic and diastolic blood pressure had a higher risk of CAD on HF. Additionally, evidence linking the causal effects of genetic liability to AF and the risk of HF was found. There was no concrete evidence linking these effects to higher heart rate or lower glomerular filtration rate (GFR). MR analysis confirmed previously found results of the causal effects of BMI as well as CAD effects for AF, BMI, and hypertension. In the GWAS, the heterogeneity in the manifestation of the diseases is likely to result in a decline of statistical power when analyzing the samples, serving as a limitation in the study. In future, other sample populations can be studied to develop a clearer understanding and to provide innovative means of treatment.

### A common variant alters SCN5A–miR-24 interaction and associates with HF mortality [123]

In this study by Zhang et al., it was found that the *SCN5A* gene is known to play a role in encoding the voltage-gated Na + channels, which play a vital role in depolarization of the cardiac action potential, as well as intercellular connection [123, 124]. Mutations in the region of the *SCN5A* sequence were found to correlate with inherited arrhythmias, cardiomyopathy as well as SNPs-associated HF-related cardiac arrest. GWAS studies have found several SNPs in the *SCN5A* locus which are tied to electrocardiographic measures and Brugada syndrome which is an inherited arrhythmic disease. In HF patients, alternatively spliced *SCN5A* transcript isoforms are associated with fatal arrhythmias. Additionally, researchers have begun describing how the *SCN5A* 3′-untranslated region (UTR) is regulated by miRs. Thus, researchers aimed to discover the underlying mechanisms of the primary cardiac voltage-gated sodium channel: Na 1.5. The expression of the SNP levels has not been thoroughly understood and studied; therefore, this study was one of the first to examine an overlooked region of miR target sites, where miR-24, which is commonly upregulated in the failing human and rodent hearts, revealed a novel and potent disease-relevant suppressor in expression of the gene.

The sample size was 1659 cardiomyopathy patients who were of European and African-American descent. To identify the miR target sites within the human myocardial tissues, the study performed high-throughput sequencing of cross-linked immunoprecipitates (HITS-CLIP). Human cardiac tissue samples were examined from the Myocardial Applied Genomics Network (MAGNet) when HF subjects underwent cardiac surgery for transplantation. Site-directed mutagenesis (New England BioLabs) was used to retrieve the C allele expression of rs1805126. Full-length *Scn5a* knockdown studies and western blot analysis were performed. To determine the correlation between miR-24 functionality and its inhibition in the sodium channels of heart cells, neonatal rat cardiomyocytes (NRCMs) were isolated from the rats and their cells were plated and cotransfected with synthetic pre-miRs, miR or siRNA expression plasmids. A few days after transfection, the Na+ currents were documented via the whole-cell patch clamp. Regarding Na+ currents, a recent study found issues such as structural derangements, mitochondrial injury, and fibrosis because of late Na+ currents in mouse hearts. GRADE and GRAPH samples and genotyping were used. LD analysis was run for the EUR and AFR populations, most relevant to the GRADE cohort. Microarray-based SCN5A was retrieved from the eQTL analyses. DHE oxidation levels in wild-type and Scn5a mouse heart tissue sections were obtained.

It was found that the regulation of the gene *SCN5A*, with SNP rs1805126, was done by miR which is a region that was often overlooked as a causal variant. Through the Ago2 HITS-CLIP, it was discovered that this area might affect an adjacent miR-24 site. It was determined that the C allele proved to be thermodynamically favorable to miR-24 suppression as opposed to the T allele. Additionally, overexpression of miR-24 inhibits Na_v_1.5 expression in human HEK293 cells therefore, affecting expression and sodium density in NRCMs as concluded by single-cell patch-clamp analyses. High levels of endogenous miR-24 expression by NRCMs pushed researchers to test whether overexpression in the miR-24 would show a correlation with rat Scn5a mRNA in NRCMs, and the results concluded that there was nearly a 50% decrease of Na_v_1.5 protein expression. When NRCMs were treated with miR-24, the expression of Na 1.5 protein decreased by nearly 50%, but the anti-miR-24 treatment had no effect on the density. In an alternate study conducted in 2021, a family across four generations with autosomal familial DCM was studied to observe the *SCN5a* variant p.C335R and concluded the loss of function in the human Na_v_1.5 channel with those possessing the variant [125]. In the experiment, iPSC-CMs were created from three individuals: one person who had the *SCN5a* variant, another individual who had both the *SCN5a* variant and TTNtv, and a final person who possessed neither of those variants. In the first group, the iPSC-CMs revealed that the mutation led to a reduction in peak Na + channels in those with the *SCN5a* variant, while those possessing both variants would experience drastic dysregulation of sarcomere structures in comparison with the other two groups. This study highlights the significance of these variants and their relationship to the DCM phenotype as well as supporting the results produced in the study conducted by Zhang et al. [123]. Researchers tested the interaction of miR-24–SCN5A SNP on human HF patients and concluded that the rs1805126 C allele was associated with adverse outcomes in HF patient cohorts. In particular, the C allele was commonly found in African-Americans and patients with a combination of CC displayed a trend of having a decrease in A-HeFT composite scores (which includes multiple factors such as death, hospitalization, and change in the quality of life). The rs1805126 C allele was found to be associated with a decrease in *SCN5A* expression in human hearts through assessment of genotype-related alteration in myocardial gene expression. Lastly, in mice, a decrease in Scn5a expression led to an accumulation of reactive oxygen species (ROS) which could possibly explain poor LV function as well as an increase in mortality of rs1805126-CC HF patients. Additionally, prior studies have reported that there is a correlation between ROS and Na_v_1.5 channel activity, and this study’s data provide evidence of elevated ROS in the hearts of Scn5a mice which confirms this relationship.

Zhang et al. did not find a correlation between the rs1805126 and PR and QRS intervals in the GRADE subjects since the measures are not definitive in HF patients due to bundle branch blocks and the intervention of pacing and biventricular pacing. Aside from the work done in this study, future studies would need to investigate other regulatory controls that have a correlation with *SCN5A* expression to determine whether the rs1805126-CC is influenced by environmental factors or other *SCN5A* SNPs. Additional research would need to confirm the role of Na 1.5 in cardiomyocytes and how ROS accumulation, can occur through components like rates of oxidative phosphorylation versus glycolysis, endosome/lysosomal acidification, or adjustment in NADPH activities.

### GWAS of left ventricular image-derived phenotypes identifies loci associated with cardiac morphogenesis and HF development [126]

This study by Aung et al. [126] aimed to identify loci that were relevant to LV imaging phenotype through GWAS using data from the UK Biobank. Since LV phenotypes play a vital role in multiple factors of CVDs including starting from diagnosis to management and risk stratification, researchers aimed to find the correlation. The sample for this study was from the UK Biobank consisting of 16,923 participants of European Ancestry whose mean age was 62.5 ± 7.5 years and consisted of 45.8% males. The data sample (including summary statistics, analytic methods, and study materials) was obtained from the UK Biobank. Genomic data was collected through the UK Biobank Axiom Array and UK BiLEVE Axiom Array to impute approximately 92 million variants. The cardiovascular magnetic resonance (CMR) phenotypes were obtained through the 1.5 Tesla scanner (MAGNETOM Aera, Syngo Platform VD13A, Siemens Healthcare, Erlangen, Germany). The primary analysis of the genetic analyses was performed by estimating the heritability and genetic correlation. Secondary analyses were performed with the adjustment of the previously performed primary analysis models and the incorporation of additional cardiovascular risk factors. Replication analysis in the MESA cohort was conducted in European and non-European ancestries. Pleiotropy analysis was performed across PubMed to obtain all significant variants genome wide. Functional annotation was combined via an integrative bioinformatics approach. PRSs of the LV phenotypes are vital to the understanding of HF since they are predictive of HF events. Ultimately, diagnosis and treatment of HF are derived from these LV functional and structural parameters.

The study found a total of 14 genome-wide significant loci associated with cardiac developmental pathways and mechanisms. There were three loci for LV end-diastolic volume (LVEDV), LV end-systolic volume (LVESV), and LV mass-to-end-diastolic volume ratio (LVMVR), four loci for LVEF, and one locus for LV mass (LVM). A total of eight genes were identified to play a role in the development of HF. The genes and SNPs included: *TTN* with SNPs rs2042995 and rs2255167 on chromosome 2, *BAG3* with SNPs rs7071853 and rs72840788 on chromosome 10, *GRK5* (a gene found in the *BAG* locus), *HSPB7*, *MTSS1* with SNP rs200712209 and rs34866937 on chromosome 8, *ALPK3* in the ZNF592 locus, *NMB* with SNPs rs2175567 and rs17598603, and *MMP11* with SNP rs2070458. Of the four loci for LVEF, the *TTN* locus was associated along with *BAG3* and *MTSS1* [127, 128]. The *BAG3* gene oversees the encoding of an antiapoptotic protein that is seen in the heart and skeletal muscle; mutations have been seen in DCM pathogenesis in myocardial tissues [127]. The three loci from LVMVR *CDKN1A*, DERL3, and *ZNF592* were not seen to associate with any other LV traits. The variants that are linked to the LV loci play a role in cardiac developmental pathways and the motion of the LV. *TTN*, *BAG3*, and *MTSS1* loci were seen in LV traits and produced results of eight unique loci and six novel ones. The loci MTSS1 and BAG3 were found to be involved in the level of Bonferroni significance and the loci for *TTN*, *SH2B3*, and *ZNF592* were also of significance.

Overall, the enrichment analyses of the study found that the variants associated with LV loci were significantly involved in cardiac developmental pathways and regulated LV contraction. Of the listed genes, several were of great importance. Firstly, the *TTN* gene is known to play a role in sarcomere assembly, signaling, and adjustment, which are all factors needed for LV imaging traits including LVEDV, LVESV, LVEF, and LVM [129]. The *BAG3* gene oversees the encoding of an antiapoptotic protein that is seen in the heart and skeletal muscle; mutations have been seen in DCM pathogenesis in myocardial tissues [127]. *MTSS1* produces a scaffold protein that controls actin dynamics and plays a role in cytoskeletal signaling [128]. Phenoscanner was used to compare the sentinel variants with other traits from previous GWASs and found that SH2B3 locus for LVEDV was correlated with multiple risk factors which include blood pressure/hypertension, cholesterol/low-density lipoprotein level, diabetes mellitus, and smoking status. Additionally, the CLCNKA and BAG3 loci were seen to play a role in the characteristic of dilated cardiomyopathy. The Gene Atlas PheWAS database found hundreds of traits in the UK Biobank with the reported sentinel variants of this study and the *SH2B3* locus was found to correlate with both hypertension and ischemic heart disease. Overall, this study combined the importance of LV phenotyping and the development of HF which may lead to the creation of novel therapeutic targets and encourage further studies in future. Although this was a GWAS, the study used a small discovery sample size that led to a < 0.5% trait variance of a few loci. The sample size did not allow the basis of the analysis to go beyond ≥ 5% of common variants with a minor allele frequency. Nevertheless, in future, the expansion of the sample size in the UK Biobank along with an improved pipeline will lead to more genetic loci discovery.

## Discussion

Through various genetic approaches, 190 genes were determined to be in association with AF (Table 1). In some cases, multiple SNPs were found in relation to a single gene, while certain genes did not have a related SNP in the literature. Genes and SNPs were discovered through both multi-ethnic as well as ancestry-specific studies, which led to the derivation of genes overlapping with two or more ethnically distinct groups. Multiple variants were found in genes responsible for, but not limited to, cardiac electrophysiology and development, cardiomyocyte contraction and structure, and the coding of sodium and potassium ion channels in the regulation of cardiac action potentials. These inherited channelopathies have a significant correlation to AF as disruption in atrial conduction and repolarization leads to atrial arrhythmia conditions demonstrated by long QT syndrome, short QT syndrome, catecholaminergic polymorphic ventricular tachycardia, and Brugada syndrome [130]. Brugada syndrome, a rare channelopathy disorder characterized by mutations in *SCN5A,* has been studied with hiPSC‐CM cell models in conjunction with the CRISPR/Cas9 system and found to be in relation to sudden cardiac arrest in adults while rarely begin diagnosed in children [131–133]. A variant in *CACNB2* causes phenotypic overlap between Brugada syndrome and short QT syndrome as well [134]. As a result, numerous phenotypes, and clinical characteristics in conjunction with AF were assessed. The more prominent being electrocardiogram parameters, hypertension, and stroke, while other conditions related to AF were peripheral vascular disease and coronary artery disease. Although AF genetic loci results were prioritized, the population cohort parameters, methodological approaches, genotyping tools, sequencing methods, and statistical analysis tools were also outlined (Table 2).

**Fig. 2:**
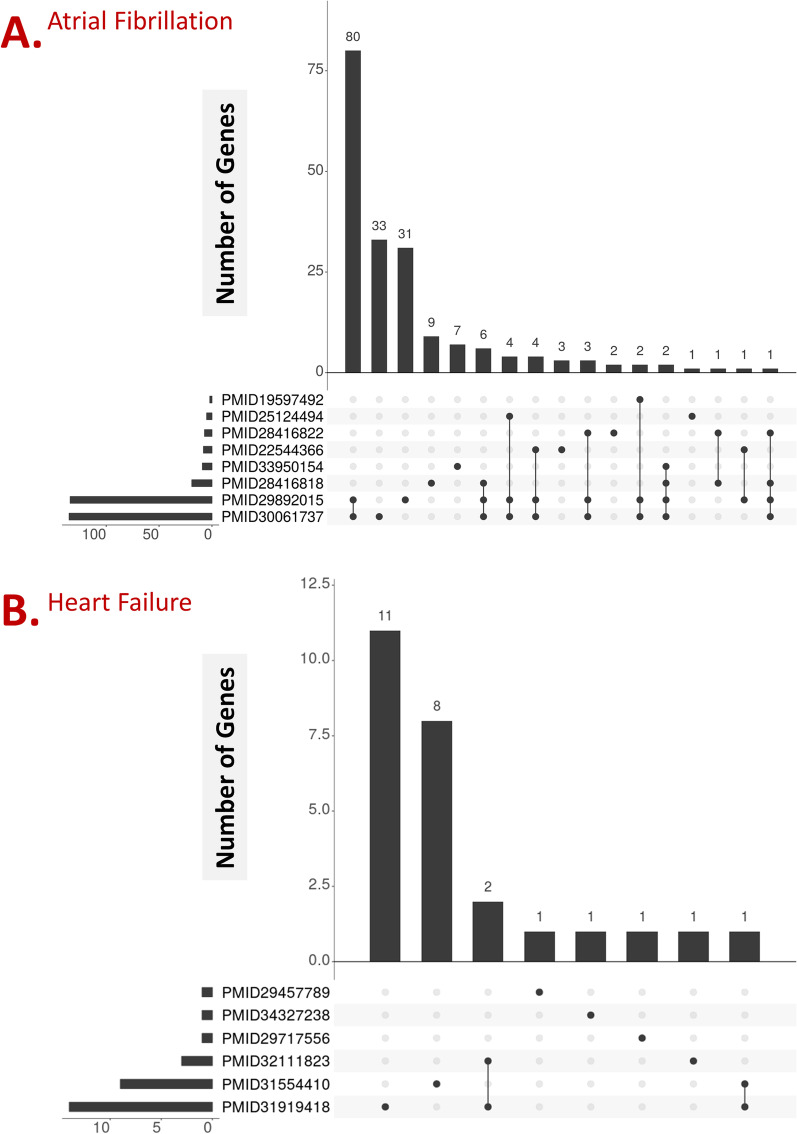
Intersection of atrial fibrillation (AF) and heart failure (HF) genes and studies reporting. The bars of the upset plot represent the unique number of genes associated with each study, while the matrix below the bars highlights the intersectionality between different studies. Dots appear to be darker and linked to genes that are included in multiple studies. The bars next to the matrix represent the cardinality of each intersection

While most AF studies conducted were two studies, both large multi-ethnic GWAS meta-analyses, utilized similar discovery cohorts that included UK Biobank and AFGen consortium [85, 94]. These two cohorts accounted for a large percentage of AF cases for both studies [85, 94]. While focusing on the discovery of novel genetic loci for AF, in some cases, previously known genes were also replicated, further validating results from other studies. Therefore, multiple genes were consistent across different studies. Figure 2A portrays the distribution and intersections of common gene findings between variable AF studies through an upset plot. The significance of the genes in relation to AF etiology increases with the level of intersection between the studies in the upset plot. *CEP68*, *TTN*, and *SCN10A* proved to be among the most prominent genes that appeared between variable studies. Likewise, a stronger correlation between genes and AF was demonstrated by the convergence of multiple SNPs on particular genes. As a result, *CAND2* (rs6810325, rs4642101, rs7650482), *PITX2* (rs2129977, rs67249485, rs17042171), *HAND2* (rs10520260, rs7698692, rs12648245), *KCNN2* (rs716845, rs337711, rs337705), *WNT8A* (rs34750263, rs2967791, rs2040862), *GJA1*(rs13191450, rs13216675, rs13195459), *NEURL* (rs11598047, rs12415501, rs6584555), and *SH3PXD2A* (rs35176054, rs202011870, rs2047036), proved to be significant as they were in association with three SNPs. Four SNPs converged to the *SLC35F1* (rs17079881, rs4946333, rs89107, rs3951016) demonstrating its importance with AF. The prominence of these genes should be further noted as the appearance in multiple studies with different genetic approaches and the convergence of numerous SNPs to the genes portray a higher correlation to AF. Concerning the unique relationship between AF genes and distinct ethnic groups, studies [35, 36, 38, 50, 78, 85, 94] used cohorts with ethnic populations to determine genes associated with AF. In various cases, it was unclear which genes and SNPs were specific to an ethnic population or shared with European populations. However, in study [36], *NEURL* was found to be in relation to both European and Japanese populations, with SNP rs1241550 for Europeans and SNP rs6584555 for Japanese populations. The findings for the relationship between *CUX2* and AF were unique to Japanese populations, while *TBX5*, *CAND2*, and an unidentified larger genetic region related to chromosome 6q22 were found to be unique to European populations. After addressing the overlap between the last two extensive GWAS meta-analyses [85, 94], most of the genes related to AF were found from combined-ancestry analysis without specification to a particular ethnic population. This includes *NEURL*, expanding the results from study [36]. Only a restricted number of genes, including *PAK2*, *GOPC*, and *CDK6*, were limited to those of European ancestry [85, 94].

A total of 26 HF-associated genes were compiled from the data presented by the articles that contributed to the overall understanding of HF (Table 3). GWAS were conducted in some of the articles to discover additional genetic variants correlated to HF. Some studies identified genes in other diseases that were observed to have been associated with cardiac features and eventual HF. Other articles used different approaches to identify genes relating to HF. However, one article examined the treatment of cancer patients with the drug doxorubicin and its correlation with DIC and HF. This study along with the others presented limitations but provided the opportunity for conducting additional research to further examine the effects of discussed genes, and the potential discovery of novel genes to aid in the overall understanding of HF. Overall, the studies concluded that the risk factors associated with HF include musculoskeletal traits, AF, body mass index, reduced left ventricular function, hypertension, and higher systolic and diastolic blood pressure equates to higher risk of coronary artery disease. The parameters chosen for all the articles include specific information regarding the type of study conducted, the population cohort parameters, sequencing methods involved, and the confirmation of such results through statistical tools and analyses (Table 4).

**Table 3:** Genes associated with heart failure (HF). This table includes information about genes, SNP, chromosome, location, and article reported

#	Gene	SNP rsID	Chromosome	Gene Location	PMID
1	*PITX2*	rs1906615; rs17042102	4	Non-coding; Intergenic	32111823; 31919418
2	*ABO*	rs9411378; rs600038, rs600038	9	Intronic; Intergenic	32111823; 31919418
3	*ACTN2*	rs580698	1	Non-coding	32111823
4	*SMAD3*	n/a	N/A	N/A	29717556
5	*MAP7D1*	rs272825; rs272832	N/A	Intronic	34327238
6	*CELSR2*	rs660240	1	UTR3	31919418
7	*FAM241A*	rs17042102	4	Intergenic	31919418
8	*KLHL3*	rs11745324	5	Intronic	31919418
9	*CDKN1A*	rs4135240	6	Intronic	31919418
10	*LPA*	rs55730499; rs140570886	6	Intronic	31919418
11	*CDKN2B-AS1*	rs1556516	9; 9p21	ncRNA	31919418
12	*SURF1*	rs600038	9	Intergenic	31919418
13	*SYNPOL2L*	rs4746140	10	Intergenic	31919418
14	*AGAP5*	rs4746140	10	Intergenic	31919418
15	*BAG3*	rs17617337; rs2234962	10	Intronic; Missense	31919418; 31554410
16	*ATXN2*	rs4766578	12	Intronic	31919418
17	*FTO*	rs5699461	16	Intronic	31919418
18	*SCN5A*	rs1805126	N/A	coding	29457789
19	*TTN*	rs2042995; rs2255167	2	N/A	31554410
20	*GRK5*	N/A	N/A	N/A	31554410
21	*HSPB7*	N/A	N/A	N/A	31554410
22	*MTSS1*	rs200712209; rs34866937; rs35006907	8	N/A	31554410
23	*ALPK3*	N/A	N/A	N/A	31554410
24	*NMB*	rs2175567; rs17598603	N/A	Intronic	31554410
25	*MMP11*	rs2070458	N/A	N/A	31554410
26	*SH2B3*	rs7310615	12	N/A	31554410

**Table 4:** Article details about genes associated with heart failure (HF)

PMID	Genes#	Samples Size	Ethnicity	Traits	P-values	NGS Type	Analysis
32111823	3	GWAS: HF = 24,829 Controls = 1,614,513	European	Ischemic heart disease: myocardial infarction, percutaneous coronary intervention or coronary artery bypass graft surgery; HF	P < 5E−8	RNA-seq	Mendelian randomization (MR Egger), linear regression
29717556	1	HF = 3	St. Louis (USA), Ghent (Belgium)	Bilateral inguinal hernia correction; retropatellar cartilage problems, verapamil for symptomatic ventricular extrasystoles	N/A	N/A	Echocardiography
34327238	1	GWAS: HF = 191	Asian, American, African, European	Cancer patients who received adjuvant doxorubicin therapy	P = 5.63E−5, P = 5.70E−5	N/A	GraphPad Prism, Linear Regression model
31919418	14	GWAS: HF = 47,309 Controls = 930,014	European	HF	p < 5E−8	RNA-seq	Two-sample MR, Mendelian randomization (MG Egger)
29457789	1	HF = 1,659	European, African-American	Cardiomyopathy patients with implantable cardioverter defibrillators (ICDs)	p < 5E−2	RNA-seq (by Illumina truSEq and Nugen Ovation)	GraphPad, Fisher's exact test, ANOVA
31554410	8	GWAS: HF = 16,923	European	Myocardial infarction or HF	1.50E−05	N/A	LDPred tool (used to obtain PRS), CMR protocol and analysis methods

This table includes information about article reported, total number of genes, sample size, ethnicity, clinical traits, P-values, next-generation sequencing (NGS) type, and analysis performed using different tools

**Fig. 3:**
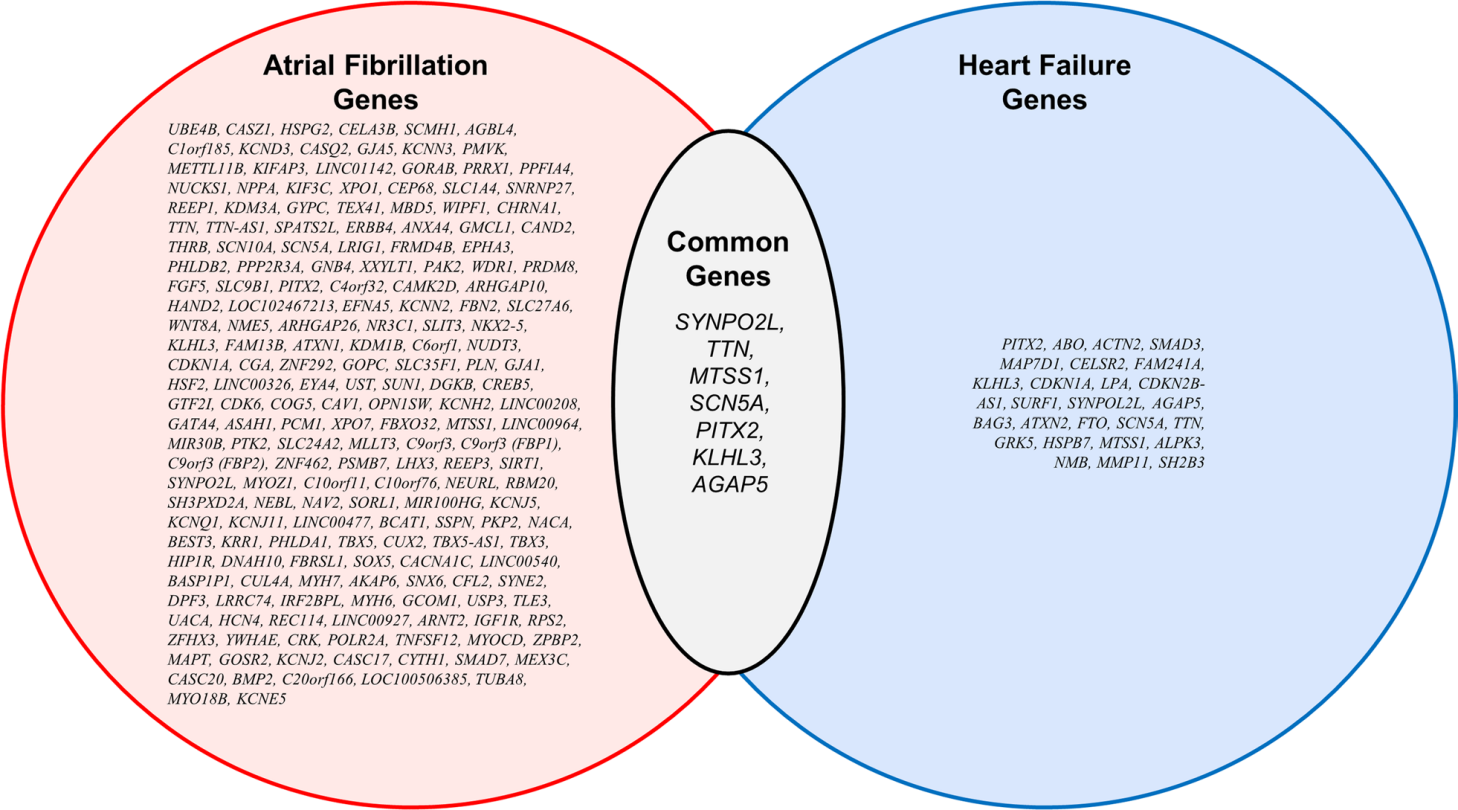
Overlapping between identified genes from different approaches for atrial fibrillation (AF) and heart failure (HF)

The HF studies analyzed were conducted with varying methods of technologies and genetic approaches. The studies primarily focused on the discovery of novel genetic loci, but some studies reported on previously discovered genes. For example, findings for *PITX2* were reported in studies [4, 117], the ABO gene was discovered in studies [4, 117], and the *BAG3* gene was reported in studies [117, 126]. However, most of the reported genes did not correlate with the other studies presented. Figure 2B details the intersection and commonality of genes across variable HF studies. *PITX2*, *ABO*, and *BAG3* demonstrate the greatest intersection between studies in the upset plot and therefore have a stronger relation to HF. Among the total HF genes, some displayed different SNPs across multiple studies which is another indication of a stronger connection to HF. *PITX2* displayed two separate SNPs (rs1906615, rs17042102) in studies [4, 117], *ABO* contained SNPs (rs9411378, rs600038) across studies [4, 117], *MAP7D1* displayed two SNPs (rs272825, rs272832) in study [115], *LPA* contained two different SNPs (rs55730499, rs140570886) in study [117], *BAG3* had two SNPs (rs17617337, rs2234962) across studies [117, 126], *TTN* had two SNPs (rs2042995, rs2255167) in a singular study [126], *MTSS1* gene noted three additional SNPs (rs200712209, rs34866937, rs35006907) in a singular study [126], and *NMB* (rs2175567, rs17598603) displayed two unique SNPs in a singular study [126]. The various SNPs reported for the different genes highlight their importance concerning HF. Most of the HF studies conducted primarily utilized a European cohort as the basis of the genetic analysis. However, studies [115, 123] included cohorts of Asians and African-Americans alongside European populations. *MAP2D1* was shared among both African-American and European cohorts. Additionally, a variant of particular importance, SNP rs1805126 C allele, portrayed a decrease in *SCN5A* expression found in African-Americans compared to other cohorts.As this study emphasized the determination of the genetic basis of CVDs, with a specification to AF and HF, the identification of seven common genes with distinct AF and HF SNPs was achieved. We reported a list of genes and their relevant SNPs that are associated with AF and HF (Table 5). The genes discussed to be associated with both AF and HF included *SYNPO2L, TTN, MTSS1, SCN5A, PITX2, KLHL3*, and *AGAP5* (Fig. 3). These genes have been reported to aid in cardiac development, control cytoskeleton signaling and encode proteins that are necessary for cardiac function. To gain a better understanding of how these seven genes impact AF and HF patients, further transcriptomic studies are necessary. Some of the limitations associated with the articles reviewed in our study include but are not limited to the absence of data regarding the lifestyle of the populations observed, restrictions on race and ethnicity of the cohort [35, 36] and small sample size [38, 78, 85, 94, 113]. Another major limitation reported by multiple articles is that the pathways associated with the identified genes are still unknown [50, 117, 123, 126]. Identifying causal genes is an essential step in translating genetic loci into biological processes. Our study aims to highlight these genes reported in recent studies, and their potential effects on molecular mechanisms for CVDs, mainly HF and AF. These studies demonstrated that combining genomic data, evaluating both common and rare genetic variants, analyzing metadata and phenotypic information, and including individuals from diverse ethnic backgrounds could provide a preliminary understanding into the identification and drug targeting of biological pathways for CVDs.

**Table 5:** List of common genes associated with atrial fibrillation (AF) and heart failure (HF)

#	Gene	Disease	Chromosome	HF SNP rsID	AF SNP rsID	Gene Location (HF)	Gene Location (AF)	Gene Role
1	*SYNPO2L*	HF, AF	10	rs4746140	rs60212594; rs10824026	intergenic	intron; 5 kb upstream	Cardiac development: expressed in skeletal and cardiac muscle, localize to the Z-disk and interact with numerous other proteins
2	*TTN*	HF, AF	2	N/A	rs35504893; rs2288327	N/A	intronic	Sarcomere assembly, stretch sensing and signaling, passive stiffness adjustment; encodes for a large sarcomeric protein known as titin
3	*MTSS1*	HF, AF	8	N/A	rs35006907	N/A	regulatory region	Cytoskeleton signaling pathway and encodes a scaffold protein which regulates actin dynamics
4	*SCN5A*	HF, AF	3	rs1805126	3 variants	coding	N/A	Associated w/expression of *MYOZ1* and *SYNPO2L* encoding 2 alpha-actinin-binding Z-disk cardiac proteins; encodes protein for cardiac sodium channel
5	*PITX2*	HF, AF	4	rs17042102	rs2129977	intergenic	intergenic	Encodes a transcription factor
6	*KLHL3*	HF, AF	5	rs11745324	rs2967791	intronic	intronic	A negative regulator of the thiazide-sensitive Na + Cl- cotransporter (*SLC12A3*) in the distal nephron
7	*AGAP5*	HF, AF	10	rs4746140	N/A	intergenic	N/A	Associated w/expression of *MYOZ1* and *SYNPO2L* encoding 2 alpha-actinin-binding Z-disk cardiac proteins

This table includes information about genes, disease, SNPs, chromosomes, location relative to gene, and gene role

Timely understanding and precise treatment of CVDs will ultimately benefit millions of individuals by reducing the high risk of mortality and improving the quality of life. Estrogens and androgens produced by gonadal hormones have been proven to play an important role for sex differences in the occurrence and progression of CVDs [135]. This difference in the manifestation of CVD between the sexes is accounted for by the roles that sex chromosomal mechanisms have in the control of diseases [135]. Causes, such as acute heart failure, reparative and interstitial fibrosis, oxidative stress, and inflammation have been linked to affecting the structural and electrophysiological remodeling of atrial tissue leading to AF [136]. AF has the potential to increase the risk factor for other diseases including, but not limited to, stroke, peripheral embolism, and Takotsubo syndrome, a condition distinguished by acute left ventricular dysfunction [136, 137]. Patients with Takotsubo syndrome who had or developed AF on admission were found to have increased mortality rates and increased hospital stays; it was shown that the in-hospital and long-term outcomes were similar whether the patient had pre-existing AF or developed AF with no prior history [138]. Similarly, patients with Takotsubo cardiomyopathy as well as AF were found to have greater mortality in both the short and long term in addition to more hospital stay discourse than those without AF [139].

Recent studies have found that CVD prognosis has improved to eight years with lifestyle changes [140, 141]. Several genetic approaches demonstrate the potential of investigating genes associated with HF, AF, and other CVDs. However, the current and still unresolved challenges include the availability of bioinformatics tools to vastly increase the performance of analysis and understand the dimensions and complexity of genomic data. GWAS has assisted in uncovering millions of loci associated with various complex phenotypes in humans, including those associated with CVDs. To further benefit from GWAS, we need systematic approaches integrating genomic data across different biological contexts to elucidate the regulatory genomic network supporting analysis of phenotypes linked to CVDs. Numerous studies have effectively utilized integrative multi-omics methods to explore new mechanisms and plasma biomarkers related to CVDs which will accelerate the discovery of novel pathways and therapeutic targets [41]. These studies demonstrate that advanced integration strategies are effective in extracting reliable biological signals across different molecular levels and phenotypes [41].

Studying genetic insight with the application of artificial intelligence (AI) and machine learning (ML), standard bioinformatics, and GWAS approaches can accelerate the processes of identifying CVD patients at increased risk of cardiac death [142–145]. These can play a vital role in the recognition, extraction, and prediction of nonlinear structures and biological patterns, which can decode the genetics of complex phenotypes. To successfully implement and achieve expected AI/ML-based results, there are challenges including missing data imputation, genomic data integration, and AI/ML ready data generation. These challenges can be addressed with the development and deployment of secure, automated, robust, intelligent, collaborative, and user-friendly frameworks for supporting personalized analyses of large and complex omics datasets in clinical settings, without requiring much bioinformatics expertise [117–119, 139]. It will provide improved and personalized treatments for HF and AF, as well as other CVDs [120], and even other complex and rare disorders. Addition of metabolomics, metatranscriptomics, and metagenomics has the potential to identify clinically significant biomarkers for disease prediction and prevention [26]. Future research endeavors may focus on analyzing diverse population-based genomics data with the nexus of standard bioinformatics and AI/ML techniques for effective translational research and provide personalized treatments to the patients with CVDs [146–148] (Additional file 1).

## 5. Conclusion

To holistically study chronic and progressive diseases with complex biological processes, it is imperative to take an integrative approach that combines genomic data to highlight the interrelationships of the involved molecular functions. Integrative genetic and clinical data analysis approaches will support diagnostic and preventive care delivery strategies beyond traditional symptom-driven and disease-causal medical practice. Detailed information associated with studies investigating genes associated with atrial fibrillation and heart failure is available in Additional file 1.

## Supplementary Information

**Additional file 1.** Detailed information associated with studies investigating genes associated with atrial fibrillation and heart failure
